# Supplementary material for: Prevalence of SARS-CoV-2 infection and immunity in a New York county in 2022 reveals frequent asymptomatic or undiagnosed infections
Source: PLoS One. 2025 May 28;20(5):e0323659. doi: 10.1371/journal.pone.0323659 (PMC12118914; doi:10.1371/journal.pone.0323659)
Supplement: S2 File — (PDF) [file pone.0323659.s002.pdf]

Consent

We are asking you to participate in a survey titled “**COVID-19 Community Surveillance Survey**”, led by Dr. Casey Cazer (Department of Public and Ecosystem Health, Cornell University College of Veterinary Medicine).

The purpose of this survey is to provide critical data on the level of COVID-19 infection and immunity in your local community, which will help public health leaders make informed decisions. This survey will take between 5 and 15 minutes.

We will ask you to provide information about your demographics, household, COVID-19 vaccination status, previous COVID-19 infections, COVID-19 prevention measures, and attitudes about COVID-19. Your household was selected at random, and **participation is voluntary. You may skip any question by clicking the 'next' button.**

Participation of children and adults with legally authorized representatives ("wards") : If you consent to your child (ages 2 and up) or ward participating in this survey and they agree, we will ask your child or ward to fill out the survey or for you to help them fill out the survey.

The **nasal swab you provided will be tested for COVID-19** by the Cornell COVID-19 Testing Laboratory in partnership with Cayuga Medical Center for COVID-19 infection by PCR, and **it will be tested for COVID-19 antibodies** by Dr. Bettina Wagner (Department of Population Medicine & Diagnostic Sciences, Cornell University College of Veterinary Medicine). **Your PCR test results will be available** through Cayuga Medical Center. Positive PCR results will be communicated to the Tompkins County Health Department. Positive samples may be sequenced, this data will NOT be returned to you. The **COVID-19 antibody results will NOT be returned to you**, Cayuga Medical Center, or the Tompkins County Health Department because the antibody test used is not FDA approved. However, a report of the aggregated data will be made publicly available.

Identifying information collected during the COVID-19 PCR test registration process will be securely stored by Cayuga Medical Center. **Identifying information is not shared with Dr. Cazer unless you opt-in to future studies**, then your contact information will be shared. **The unique code on your nasal swab tube will be used to link the COVID-19 test results and your anonymous survey answers**. Despite these measures, we cannot guarantee anonymity of your personal data. The anonymous nasal swab sample and survey information may be used in future studies and shared with other researchers.

If you have questions, please contact Dr. Cazer at casey.cazer@cornell.edu or 607-253-4086. This project is paid for by the David and Lucile Packard Foundation and is supported by the Tompkins County Health Department and Cayuga Health Partners

Proceeding to the survey indicates your consent to participate or your consent for your child or ward to participate.

Demographics

Sample ID.

If there is no number listed, please check with the Field Assistants.

DEMOGRAPHICS

These questions ask about your demographics. Some information may have been pre-filled based on your COVID-19 test registration. Please confirm that this information is correct.

Year of birth

Gender

- ○ Male
- ○ Female
- ○ Transgender
- ○ Gender non-binary
- ○ Other

Race (select one or more)

- ☐ American Indian or Alaskan Native
- ☐ Native Hawaiian or Pacific Islander
- ☐ Asian
- ☐ White
- ☐ Black or African American

Ethnicity

- ○ Hispanic or Latino
- ○ Not Hispanic or Latino

Education & Employment

Which of the following describes your highest education level completed?

- ○ none or between grades 1 - 8
- ○ Between grades 9 - 11
- ○ High School Diploma / GED certificate
- ○ Technical, trade or vocational school
- ○ Associate's Degree
- ○ Bachelor's Degree

○ Graduate/ Professional School Degree

Which of the following best describes your current employment status? (select all that apply)

☐ Full time employment
☐ Part time employment
☐ Self-employed
☐ Unemployed
☐ Disabled
☐ Student
☐ Retired

What best describes how you currently work?

○ All in-person (on-site) work
○ Mostly in-person (more than half) with some remote work
○ About half in-person and half remote
○ Mostly remote (more than half) with some in-person work
○ All remote work
○ Other (describe): ________

When you are working in-person, how many people do you have close contact with on a typical work day?

Close contact is defined as being within 6 ft for at least 15 minutes.

○ 0 to 5
○ 6 to 10
○ 11 to 20
○ More than 20

When you are working in-person, do you interact with members of the public on a typical work day?

- Yes
- No

Household

HOUSEHOLD

These next questions ask about your household. A household is defined as a housing unit such as a home, apartment, or other group of rooms that are occupied by an individual or group of people, including family related by birth, marriage or adoption, and/or non-family such as foster children or unrelated roommates sharing the same living quarters. A household does not include temporary visitors.

How many people, including yourself, are currently living in your household? This includes all people related and unrelated sharing the same living quarters but does not include temporary visitors.

What is the age (in years as of their last birthday) of the youngest person in your household?

What is the age (in years as of their last birthday) of the oldest person in your household?

Vaccination

VACCINATION

These next questions ask about COVID-19 vaccination.

Are you vaccinated against COVID-19?

- ○ Yes
- ○ No
- ○ Partially (one of the two-shot series of the Pfizer, Moderna or other vaccines)

What are the reasons that you are not vaccinated against COVID-19 yet? (select all that apply)

- ☐ too young to get vaccinated
- ☐ don't think COVID-19 is a threat
- ☐ don't trust government agencies (CDC, Health Departments)
- ☐ religious reasons
- ☐ don't think the vaccine is effective
- ☐ difficulty accessing a vaccination clinic
- ☐ don't trust the companies manufacturing the vaccines
- ☐ don't trust the scientists developing the vaccines
- ☐ severe allergic reaction to vaccines
- ☐ concerns about vaccine side effects
- ☐ Other (describe) ______

Do you plan to get vaccinated in the next 30 days?

- ○ Yes
- ○ No
- ○ Unsure

Would you like someone in the health department to contact you with additional information about getting vaccinated, and to answer any questions you might have?

- ○ Yes
- ○ No

What vaccine(s) did you receive for your primary vaccination series (excluding any boosters)??

- ☐ Pfizer-BioNTech
- ☐ Moderna
- ☐ Johnson & Johnson
- ☐ Other (please type name in box) ________
- ☐ Unsure

When did you receive your second shot in the two-dose series, or your first shot if only one was required?

Month ▾

Year ▾

When did you receive your first shot?

Month ▾

Year ▾

Have you received a COVID-19 booster vaccine?

- ○ Yes, one booster
- ○ Yes, more than one booster
- ○ No

What booster vaccine did you receive most recently?

- ○ Pfizer-BioNTech
- ○ Moderna
- ○ Johnson & Johnson
- ○ Other (please type name in box) ______
- ○ Unsure

When did you receive your most recent booster shot?

Month [ ]

Year [ ]

Quater done

You have finished about one quarter of the survey.

COVID-19 Infections

COVID-19.

These next questions ask about COVID-19 infections and exposure.

Have you ever **tested positive** for COVID-19?

- ○ Yes
- ○ No

How many times have you had COVID-19?

When did you test positive for COVID-19?

If you had COVID-19 more than once, give the most recent test positive date.

Month

Year

Do you **think** you had COVID-19 even though you never tested positive?

- Yes
- No
- Unsure

When do you think you had COVID-19?

Month

Year

Have any of your family or friends tested positive for COVID-19?

- Yes
- No

Throughout the pandemic, were you ever in **mandatory isolation** or in **mandatory quarantine** as determined by your local health department?

In general, the Health Department sends **isolation** notifications to individuals who test positive for COVID-19 and the Health Department sends **quarantine** notifications to individuals who were exposed to someone infected with COVID-19.

- Yes, I was in mandatory isolation for testing positive.

○ Yes, I was in mandatory quarantine for being exposed.

○ Yes, I was in mandatory isolation for testing positive AND in mandatory quarantine for being exposed.

○ No, I was never in mandatory isolation nor mandatory quarantine.

○ Unsure

Isolation and Quarantine

When you were in mandatory isolation and/or mandatory quarantine, did you have any difficulties obtaining anything needed to support your health and your household? Check all that apply.

☐ COVID-19 testing

☐ COVID-19 test kit delivery to your home

☐ Prescription medications

☐ Over-the-counter medications

☐ Thermometer

☐ Cleaning supplies

☐ 211 call-line food boxes

☐ Food delivery

☐ Other (describe in box): ________

☐ I didn't have any difficulties getting needed items

Please describe the type of difficulties you had meeting your needs during isolation/quarantine in the space below.

____

When you were in mandatory isolation or mandatory quarantine, did you make use of any community services or other social services? (check all that apply)

☐ Mutual Aid Tompkins

- [ ] Medicaid or Medicaid Taxi
- [ ] Mental health support
- [ ] Funding through the Family Medical Leave Act [FMLA]
- [ ] 211 county referral call line
- [ ] REACH Medical
- [ ] Tompkins Community Action
- [ ] Other(s)--(Describe in box): ____________
- [ ] I didn't use any community or social services

We have all been affected by COVID-19. Did you have any major changes in your life since the start of the COVID-19 pandemic (around February of 2020)? Select all that apply.

- [ ] New job
- [ ] Enrolled in public services for the first time
- [ ] Lost housing
- [ ] Moved within Tompkins County
- [ ] Could not afford childcare
- [ ] Could not afford housing
- [ ] Lost employment
- [ ] Lost childcare
- [ ] Loss of a loved one to COVID-19
- [ ] Moved from a different county, state, or country to Tompkins County
- [ ] Other(s)--(describe in box): ____________
- [ ] I didn't have any major life changes

General Health

GENERAL HEALTH.

These next few questions ask some general questions about your health and smoking/vaping habits.

In the **past 7 days**, have you been experiencing any of the following symptoms?

Please select all symptoms that apply, even if you don't think they are due to COVID-19.

- ☐ Sore throat
- ☐ Cough
- ☐ Fever or chills
- ☐ Headache
- ☐ Congestion or runny nose
- ☐ Fatigue
- ☐ Diarrhea
- ☐ Muscle or body aches
- ☐ New loss of taste or smell
- ☐ Nausea or vomiting
- ☐ Shortness of breath or difficulty breathing
- ☐ Other (describe in box): ____________
- ☐ No symptoms at all

Do you think that your health puts you at high risk of severe COVID-19 illness?

- ○ Yes
- ○ No
- ○ Unsure

Did you have physical or mental health problems that lasted for **4 or more weeks** after your COVID-19 infection?

- ○ Yes
- ○ No
- ○ Unsure

Smoking

How would you characterize your tobacco smoking status?

- ○ current smoker
- ○ former smoker
- ○ never smoked

How would you characterize your vaping status?

- ○ current user
- ○ former user
- ○ never used a vaping device

Half done

You have finished about half of the survey.

Behaviors

PRACTICES, BELIEFS AND ATTITUDES.

The next set of questions will ask you about your practices, beliefs and attitudes about COVID-19 precautions and activities such as mask-wearing, social distancing, travel, attendance at smaller gatherings and larger events.

In the **last two weeks**, how often have you **worn a mask** indoors in public places?

- ○ Never
- ○ Less than half of the time
- ○ About half of the time

- More than half of the time
- Always
- I don't go to public places

What type of mask(s) do you usually wear (check all that apply)?

- Cloth
- Surgical/Medical
- Respirator (such as N95, KN95, FFP2, KF94)
- Other (describe in box):
- I never wear a mask

In the **last two weeks**, how often have you **maintained physical distance** (6 feet apart from other people) indoors in public places?

- Never
- Less than half of the time
- About half of the time
- More than half of the time
- Always
- I don't go to public places

In the **last two weeks**, how often have you used any of these forms of public transportation? (check one circle for each type of transport)

	not at all	once	twice	3-5 times	6-10 times	more than 10 times
TCAT, public bus, or school bus	○	○	○	○	○	○
Airplane	○	○	○	○	○	○
Train	○	○	○	○	○	○
Metro/subway	○	○	○	○	○	○

	not at all	once	twice	3-5 times	6-10 times	more than 10 times
Uber, Lyft, cab or other ride-sharing service	○	○	○	○	○	○

In the **last two weeks,** where have you traveled outside Tompkins County? (check all that apply)

- ☐ A neighboring county in NY State
- ☐ Somewhere else in NY State (not a neighboring county)
- ☐ Another State
- ☐ International
- ☐ I have not traveled outside Tompkins County

Please choose the reason(s) that best describe why you decided to travel in the **last two weeks**. (check all that apply)

- ☐ Medical
- ☐ Family need
- ☐ For current school or work needs
- ☐ For fun; to get away
- ☐ Other (describe in the box): ____________

In the **last two weeks**, have you attended any indoor in-person social gatherings, not counting those at work or school?

- ○ Yes
- ○ No

How many indoor in-person social gatherings have you attended in the **last two weeks**, not counting those at work or school?

____

At the indoor in-person social gathering(s), did you **wear a mask**?

- Never
- Less than half of the time
- About half of the time
- More than half of the time
- Always (except to eat or drink)
- My answer depends upon the event I attended. (Explain in the box below)

At the indoor in-person social gatherings, how often have you **maintained physical distance** (6 feet apart from other people)?

- Never
- Less than half of the time
- About half of the time
- More than half of the time
- Always
- My answer depends upon the event I attended. (Explain in the box below)

In the **last two weeks**, have you attended any large events (more than 100 people)? For example, a concert, performance, sporting event, festival, or large wedding?

- Yes
- No

How many large events (more than 100 people) have you attended **in the last two weeks**? For example, a concert, performance, sporting event, festival, or large wedding.

Where did the large event(s) take place?

- ◯ Entirely outdoors
- ◯ Entirely indoors
- ◯ Some events, or parts of an event, were indoors and some were outdoors

In the **last two weeks**, how many times have you gone to a gym/fitness center?

- ◯ 0
- ◯ 1 - 3 times
- ◯ 4 - 6 times
- ◯ 7 - 9 times
- ◯ 10 - 12 times
- ◯ more than 12 times

In the **last two weeks**, how many times have you eaten indoors in restaurants?

- ◯ 0
- ◯ 1 - 3 times
- ◯ 4 - 6 times
- ◯ 7 - 9 times
- ◯ More than 9 times

Almost done

You are almost done with the survey!

Attitudes

Compared to before the COVID-19 pandemic, have you changed the frequency of these behaviors?

	Increased this behavior	Haven't changed	Decreased this behavior
Going to the doctor's or dentist's office	○	○	○
Curbside pickup/take-out	○	○	○
Use of telehealth services	○	○	○
Cleaning/disinfecting surfaces	○	○	○
Touching eyes or mouth	○	○	○
Use of hand sanitizers	○	○	○
Hand-washing	○	○	○
Visiting nursing homes	○	○	○
Staying home when sick	○	○	○

Physical distancing is important to reduce the risk of COVID-19 for myself and others.

- ○ Strongly disagree
- ○ Disagree
- ○ Neither agree nor disagree
- ○ Agree
- ○ Strongly agree

Wearing a mask is important to reduce the risk of COVID-19 for myself and others.

- ○ Strongly disagree
- ○ Disagree
- ○ Neither agree nor disagree
- ○ Agree
- ○ Strongly agree

It is important to **minimize travel** to reduce the risk of COVID-19 transmission.

- ◯ Strongly disagree
- ◯ Disagree
- ◯ Neither agree nor disagree
- ◯ Agree
- ◯ Strongly agree

How worried are you about getting COVID-19 **in the next three months**?

- ◯ Very worried
- ◯ Somewhat worried
- ◯ Not that worried
- ◯ Not at all worried

When the number of COVID-19 cases is high in Tompkins County, I am more focused on following public health protocols.

- ◯ Strongly disagree
- ◯ Disagree
- ◯ Neither agree nor disagree
- ◯ Agree
- ◯ Strongly agree

Where do you get information pertaining to the number of COVID-19 cases in Tompkins County? (check all that apply)

- ☐ Ithaca Voice Dashboard
- ☐ Tompkins County Public Health Department
- ☐ NYS Forward
- ☐ Other (describe in box): ________
- ☐ I don't look up the number of COVID-19 cases

How do you prefer to receive information from the Tompkins County Health Department about COVID-19? (check all that apply)

- ☐ Email
- ☐ Website
- ☐ Social media
- ☐ 211 referral line
- ☐ Robo calls
- ☐ Text messages
- ☐ Other (describe in box): ____________

Follow up

Thank you for your participation! A lot of important scientific and public health questions about COVID-19 are still unanswered. May we contact you with opportunities to participate in follow-up studies that can answer questions about COVID-19 transmission and immunity?

If yes, we may reach out to you using the phone number or email you provided for the PCR test registration.

- ○ Yes
- ○ No

Powered by Qualtrics
