# Supplementary material for: Prevalence of SARS-CoV-2 infection and immunity in a New York county in 2022 reveals frequent asymptomatic or undiagnosed infections
Source: PLoS One. 2025 May 28;20(5):e0323659. doi: 10.1371/journal.pone.0323659 (PMC12118914; doi:10.1371/journal.pone.0323659)
Supplement: S4 File — (DOCX) [file pone.0323659.s004.docx]

**Cayuga Health System Off-Line Patient Registration for Community Surveillance Study Participants**

**Date of Registration/Sample Collection:** __________________

It is important that you confirm that the ID below is the same ID that appears on your sample and also that you are making this registration on the same date that you have created the sample.

**Sample / Barcode ID:** _____________

**Sponsoring organization:** Community Surveillance Study

**Affiliation to Organization / Employer:** OTHER.

**Collection Method:** Anterior Nares swab

**Questions (Circle either Yes or No):**

1. Are you a First Responder, Healthcare Worker, or other Essential Worker? **Yes / No**
2. Have you been diagnosed with COVID-19 or received a positive test for COVID-19 in the past? **Yes / No**

**Demographics * = Required; ** = Recommended**

First Name* _________________________

Last Name* _________________________

Birthdate* Month/Day/Year following this format:

mm/dd/yyyy

_ _/_ _/_ _ _ _

Gender

__ Male

__ Female

__ Transgender

__ Gender non-binary

__ Other

Race—Select one or more

__ American Indian or Alaskan Native

__ Native Hawaiian or Pacific Islander

__ Asian

__ White

__ Black or African American

Ethnicity

__ Hispanic or Latino

__ Not Hispanic or Latino

Cell Phone * (_ _ _) _ _ _-_ _ _ _

(Area code)

Home Phone (_ _ _) _ _ _-_ _ _ _

(Area code)

**Page 1 of 2.**

Email Address** ________________________________

**Local Address**

Address* ______________________________________

City* Ithaca

State* New York

Zip Code* ______________________________________

**Acknowledgement, Consent, & Release**

**Acknowledgement & Consent**

I hereby authorize Cayuga Health System and its affiliates, including Cayuga Medical Center and Cayuga Medical Associates, to examine, diagnose, and assess my health conditions, and to provide services to effectively treat me. I understand that health information about me may be shared for treatment of my condition, payment for services provided, and normal business operations. For these purposes, we may disclose your information to other healthcare providers including: pathologists, radiologist, and emergency physicians. I acknowledge and agree that this applies to all visits at Cayuga Health System and its affiliates, including Cayuga Medical Center and Cayuga Medical Associates. I acknowledge that I have been offered the HIPAA Notice of Privacy Practices and that I am duly authorized to provide acknowledgement of receipt. Notice of Privacy Practices are available at <https://www.cayugamedicalassociates.org/notice-of-privacy-policy/> and <https://www.cayugamed.org/protecting-patient-privacy/> .

I acknowledge that I have read and understand the above information.

**Release of Information**

I hereby authorize Cayuga Health System and its affiliates, including Cayuga Medical Center and Cayuga Medical Associates to release copies of all of my COVID-19 testing, screening, vaccination, and consult records and demographic information to the organization or school identified at the beginning of this registration. Unless it is revoked, this authorization will remain in effect until 180 days after the end of the COVID-19 Public Health Emergency declared or renewed by US Department of Health and Human Services. I understand I may revoke this authorization at any time by presenting written revocation to the Health Information Management Department of Cayuga Medical Center. Revocation will not apply to information already released in response to this authorization. I understand that any release of information carries with it the potential for re-disclosure by the recipient and may not be protected by the federal privacy rules. Cayuga Medical Center and Cayuga Medical Associates will not condition treatment, payment, or eligibility of benefits on completion of an authorization. You may request a copy of this authorization.

**Signature *: _______________________________________________**

**If patient representative, relationship to patient: _______________________________________________**

**Page 2 of 2.**
