# Supplementary material for: Prevalence of SARS-CoV-2 infection and immunity in a New York county in 2022 reveals frequent asymptomatic or undiagnosed infections
Source: PLoS One. 2025 May 28;20(5):e0323659. doi: 10.1371/journal.pone.0323659 (PMC12118914; doi:10.1371/journal.pone.0323659)
Supplement: S9 Table — Table of the univariate comparisons between antibody presence and attitude risk factors for infection in April 2022. (HTML) [file pone.0323659.s009.html]

| **Characteristic** | **N Missing** | **Overall** N=471 | **FALSE** N=271 | **TRUE** N=201 | **p-value**2 |
| --- | --- | --- | --- | --- | --- |
| DistanceImportant | 1 |  |  |  | 0.433 |
| Strongly agree |  | 24 (52%) | 11 (47%) | 13 (59%) |  |
| Agree |  | 15 (34%) | 11 (38%) | 4 (28%) |  |
| Neither agree nor disagree |  | 2 (3.2%) | 1 (2.1%) | 1 (4.6%) |  |
| Disagree |  | 1 (2.0%) | 0 (0%) | 1 (4.6%) |  |
| Strongly disagree |  | 4 (8.6%) | 3 (13%) | 1 (3.1%) |  |
| MaskImportant | 1 |  |  |  | 0.006 |
| Strongly agree |  | 30 (63%) | 13 (48%) | 17 (82%) |  |
| Agree |  | 9 (18%) | 7 (21%) | 2 (13%) |  |
| Neither agree nor disagree |  | 3 (9.3%) | 3 (17%) | 0 (0%) |  |
| Disagree |  | 1 (2.0%) | 0 (0%) | 1 (4.6%) |  |
| Strongly disagree |  | 3 (7.9%) | 3 (14%) | 0 (0%) |  |
| TravelImportant | 1 |  |  |  | 0.052 |
| Strongly agree |  | 12 (24%) | 6 (20%) | 6 (28%) |  |
| Agree |  | 20 (46%) | 9 (36%) | 11 (59%) |  |
| Neither agree nor disagree |  | 9 (21%) | 8 (34%) | 1 (4.6%) |  |
| Disagree |  | 3 (5.4%) | 2 (6.0%) | 1 (4.6%) |  |
| Strongly disagree |  | 2 (3.4%) | 1 (3.6%) | 1 (3.1%) |  |
| Worried | 1 |  |  |  | 0.988 |
| Not at all worried |  | 7 (14%) | 4 (13%) | 3 (14%) |  |
| Not that worried |  | 16 (35%) | 8 (30%) | 8 (41%) |  |
| Somewhat worried |  | 20 (45%) | 14 (57%) | 6 (29%) |  |
| Very worried |  | 3 (6.8%) | 0 (0%) | 3 (15%) |  |
| FollowProtocols | 1 |  |  |  | 0.380 |
| Strongly agree |  | 18 (40%) | 9 (35%) | 9 (46%) |  |
| Agree |  | 19 (41%) | 11 (41%) | 8 (41%) |  |
| Neither agree nor disagree |  | 3 (5.7%) | 3 (10%) | 0 (0%) |  |
| Disagree |  | 1 (2.7%) | 1 (4.8%) | 0 (0%) |  |
| Strongly disagree |  | 5 (11%) | 2 (9.3%) | 3 (12%) |  |
| DistanceImportant2 | 1 |  |  |  | 0.798 |
| Agree |  | 39 (86%) | 22 (85%) | 17 (88%) |  |
| Neither agree nor disagree |  | 2 (3.2%) | 1 (2.1%) | 1 (4.6%) |  |
| Disagree |  | 5 (11%) | 3 (13%) | 2 (7.7%) |  |
| MaskImportant2 | 1 |  |  |  | 0.057 |
| Agree |  | 39 (81%) | 20 (69%) | 19 (95%) |  |
| Neither agree nor disagree |  | 3 (9.3%) | 3 (17%) | 0 (0%) |  |
| Disagree |  | 4 (9.9%) | 3 (14%) | 1 (4.6%) |  |
| TravelImportant2 | 1 |  |  |  | 0.022 |
| Agree |  | 32 (70%) | 15 (56%) | 17 (88%) |  |
| Neither agree nor disagree |  | 9 (21%) | 8 (34%) | 1 (4.6%) |  |
| Disagree |  | 5 (8.8%) | 3 (9.6%) | 2 (7.7%) |  |
| FollowProtocols2 | 1 |  |  |  | 0.328 |
| Agree |  | 37 (81%) | 20 (76%) | 17 (88%) |  |
| Neither agree nor disagree |  | 3 (5.7%) | 3 (10%) | 0 (0%) |  |
| Disagree |  | 6 (13%) | 3 (14%) | 3 (12%) |  |
|  |  |  |  |  |  |
| --- | --- | --- | --- | --- | --- |
| 1 n unweighted (% weighted) | | | | | |
| 2 Wilcoxon rank-sum test for complex survey samples | | | | | |
