# Supplementary material for: Prevalence of SARS-CoV-2 infection and immunity in a New York county in 2022 reveals frequent asymptomatic or undiagnosed infections
Source: PLoS One. 2025 May 28;20(5):e0323659. doi: 10.1371/journal.pone.0323659 (PMC12118914; doi:10.1371/journal.pone.0323659)
Supplement: S10 Table — Table of the univariate comparisons between antibody presence and behavior risk factors for infection in April 2022. (HTML) [file pone.0323659.s010.html]

| **Characteristic** | **N Missing** | **Overall** N=471 | **FALSE** N=271 | **TRUE** N=201 | **p-value**2 |
| --- | --- | --- | --- | --- | --- |
| Smoking | 1 |  |  |  | 0.015 |
| current smoker |  | 2 (4.3%) | 1 (2.9%) | 1 (6.2%) |  |
| former smoker |  | 13 (29%) | 4 (11%) | 9 (52%) |  |
| never smoked |  | 31 (66%) | 21 (86%) | 10 (42%) |  |
| Smoking2 | 0 |  |  |  | 0.021 |
|  |  | 1 (3.9%) | 1 (6.7%) | 0 (0%) |  |
| current or former smoker |  | 15 (32%) | 5 (13%) | 10 (58%) |  |
| never smoked |  | 31 (64%) | 21 (80%) | 10 (42%) |  |
| Vaping | 1 |  |  |  | 0.322 |
| current user |  | 0 (0%) | 0 (0%) | 0 (0%) |  |
| former user |  | 2 (5.4%) | 0 (0%) | 2 (12%) |  |
| never used a vaping device |  | 44 (95%) | 26 (100%) | 18 (88%) |  |
| Vaping2 | 0 |  |  |  | 0.368 |
|  |  | 1 (3.9%) | 1 (6.7%) | 0 (0%) |  |
| current or former vaper |  | 2 (5.2%) | 0 (0%) | 2 (12%) |  |
| never used a vaping device |  | 44 (91%) | 26 (93%) | 18 (88%) |  |
| Masking | 1 |  |  |  | 0.163 |
| I don't go to public places |  | 0 (0%) | 0 (0%) | 0 (0%) |  |
| Always |  | 19 (43%) | 9 (34%) | 10 (55%) |  |
| More than half of the time |  | 12 (28%) | 8 (34%) | 4 (20%) |  |
| About half of the time |  | 5 (10%) | 3 (11%) | 2 (9.9%) |  |
| Less than half of the time |  | 7 (13%) | 4 (14%) | 3 (11%) |  |
| Never |  | 3 (6.1%) | 2 (7.2%) | 1 (4.6%) |  |
| Masking2 | 1 |  |  |  | 0.638 |
| Less than half of the time |  | 10 (19%) | 6 (21%) | 4 (16%) |  |
| About half of the time |  | 5 (10%) | 3 (11%) | 2 (9.9%) |  |
| More than half of the time |  | 31 (71%) | 17 (68%) | 14 (75%) |  |
| MaskType | 1 |  |  |  | 0.014 |
| Cloth |  | 3 (6.0%) | 3 (11%) | 0 (0%) |  |
| Cloth,Other (describe in box): |  | 0 (0%) | 0 (0%) | 0 (0%) |  |
| Cloth,Respirator (such as N95, KN95, FFP2, KF94) |  | 0 (0%) | 0 (0%) | 0 (0%) |  |
| Cloth,Surgical/Medical |  | 3 (6.8%) | 2 (7.2%) | 1 (6.2%) |  |
| Cloth,Surgical/Medical,Other (describe in box): |  | 0 (0%) | 0 (0%) | 0 (0%) |  |
| Cloth,Surgical/Medical,Respirator (such as N95, KN95, FFP2, KF94) |  | 3 (5.0%) | 1 (2.9%) | 2 (7.7%) |  |
| I never wear a mask |  | 0 (0%) | 0 (0%) | 0 (0%) |  |
| Respirator (such as N95, KN95, FFP2, KF94) |  | 24 (57%) | 14 (57%) | 10 (56%) |  |
| Surgical/Medical |  | 9 (16%) | 2 (5.7%) | 7 (30%) |  |
| Surgical/Medical,Other (describe in box): |  | 0 (0%) | 0 (0%) | 0 (0%) |  |
| Surgical/Medical,Respirator (such as N95, KN95, FFP2, KF94) |  | 4 (9.3%) | 4 (17%) | 0 (0%) |  |
| MaskType2 | 1 |  |  |  | 0.496 |
| I never wear a mask |  | 0 (0%) | 0 (0%) | 0 (0%) |  |
| Cloth |  | 3 (6.0%) | 3 (11%) | 0 (0%) |  |
| Surgical/Medical |  | 12 (23%) | 4 (13%) | 8 (36%) |  |
| Respirator |  | 31 (71%) | 19 (76%) | 12 (64%) |  |
| Distancing | 1 |  |  |  | 0.932 |
| I don't go to public places |  | 0 (0%) | 0 (0%) | 0 (0%) |  |
| Always |  | 6 (16%) | 3 (11%) | 3 (22%) |  |
| More than half of the time |  | 14 (27%) | 8 (30%) | 6 (25%) |  |
| About half of the time |  | 9 (24%) | 7 (32%) | 2 (14%) |  |
| Less than half of the time |  | 13 (26%) | 5 (18%) | 8 (35%) |  |
| Never |  | 4 (7.2%) | 3 (9.3%) | 1 (4.6%) |  |
| Distancing2 | 1 |  |  |  | 0.884 |
| Less than half of the time |  | 17 (33%) | 8 (27%) | 9 (40%) |  |
| About half of the time |  | 9 (24%) | 7 (32%) | 2 (14%) |  |
| More than half of the time |  | 20 (43%) | 11 (40%) | 9 (46%) |  |
| Bus | 1 |  |  |  | 0.316 |
| not at all |  | 38 (82%) | 20 (76%) | 18 (88%) |  |
| once |  | 1 (1.6%) | 1 (2.9%) | 0 (0%) |  |
| twice |  | 2 (5.2%) | 1 (2.1%) | 1 (9.2%) |  |
| 3-5 times |  | 3 (7.9%) | 2 (12%) | 1 (2.6%) |  |
| 6-10 times |  | 0 (0%) | 0 (0%) | 0 (0%) |  |
| more than 10 times |  | 2 (3.7%) | 2 (6.5%) | 0 (0%) |  |
| Bus2 | 1 |  |  |  | 0.341 |
| not at all |  | 38 (82%) | 20 (76%) | 18 (88%) |  |
| 1-5 times |  | 6 (15%) | 4 (17%) | 2 (12%) |  |
| More than 5 times |  | 2 (3.7%) | 2 (6.5%) | 0 (0%) |  |
| Plane | 2 |  |  |  | 0.678 |
| not at all |  | 37 (80%) | 20 (77%) | 17 (83%) |  |
| once |  | 3 (7.4%) | 2 (9.6%) | 1 (4.6%) |  |
| twice |  | 5 (13%) | 3 (14%) | 2 (12%) |  |
| 3-5 times |  | 0 (0%) | 0 (0%) | 0 (0%) |  |
| 6-10 times |  | 0 (0%) | 0 (0%) | 0 (0%) |  |
| more than 10 times |  | 0 (0%) | 0 (0%) | 0 (0%) |  |
| Plane2 | 2 |  |  |  | 0.655 |
| not at all |  | 37 (80%) | 20 (77%) | 17 (83%) |  |
| 1-5 times |  | 8 (20%) | 5 (23%) | 3 (17%) |  |
| More than 5 times |  | 0 (0%) | 0 (0%) | 0 (0%) |  |
| Train | 2 |  |  |  | 0.238 |
| not at all |  | 43 (95%) | 25 (100%) | 18 (88%) |  |
| once |  | 1 (1.2%) | 0 (0%) | 1 (2.6%) |  |
| twice |  | 1 (4.1%) | 0 (0%) | 1 (9.2%) |  |
| 3-5 times |  | 0 (0%) | 0 (0%) | 0 (0%) |  |
| 6-10 times |  | 0 (0%) | 0 (0%) | 0 (0%) |  |
| more than 10 times |  | 0 (0%) | 0 (0%) | 0 (0%) |  |
| Train2 | 2 |  |  |  | 0.235 |
| not at all |  | 43 (95%) | 25 (100%) | 18 (88%) |  |
| 1-5 times |  | 2 (5.3%) | 0 (0%) | 2 (12%) |  |
| More than 5 times |  | 0 (0%) | 0 (0%) | 0 (0%) |  |
| Metro | 4 |  |  |  | 0.369 |
| not at all |  | 40 (93%) | 23 (89%) | 17 (97%) |  |
| once |  | 1 (1.7%) | 1 (3.0%) | 0 (0%) |  |
| twice |  | 0 (0%) | 0 (0%) | 0 (0%) |  |
| 3-5 times |  | 1 (1.2%) | 0 (0%) | 1 (2.9%) |  |
| 6-10 times |  | 1 (4.3%) | 1 (7.5%) | 0 (0%) |  |
| more than 10 times |  | 0 (0%) | 0 (0%) | 0 (0%) |  |
| Metro2 | 4 |  |  |  | 0.363 |
| not at all |  | 40 (93%) | 23 (89%) | 17 (97%) |  |
| 1-5 times |  | 2 (3.0%) | 1 (3.0%) | 1 (2.9%) |  |
| More than 5 times |  | 1 (4.3%) | 1 (7.5%) | 0 (0%) |  |
| Cab | 6 |  |  |  | 0.619 |
| not at all |  | 35 (86%) | 22 (89%) | 13 (82%) |  |
| once |  | 2 (4.5%) | 0 (0%) | 2 (11%) |  |
| twice |  | 2 (2.8%) | 0 (0%) | 2 (6.8%) |  |
| 3-5 times |  | 2 (6.4%) | 2 (11%) | 0 (0%) |  |
| 6-10 times |  | 0 (0%) | 0 (0%) | 0 (0%) |  |
| more than 10 times |  | 0 (0%) | 0 (0%) | 0 (0%) |  |
| Cab2 | 6 |  |  |  | 0.527 |
| not at all |  | 35 (86%) | 22 (89%) | 13 (82%) |  |
| 1-5 times |  | 6 (14%) | 2 (11%) | 4 (18%) |  |
| More than 5 times |  | 0 (0%) | 0 (0%) | 0 (0%) |  |
| Travel | 1 |  |  |  | 0.309 |
| A neighboring county in NY State |  | 6 (11%) | 1 (2.9%) | 5 (22%) |  |
| A neighboring county in NY State,Another State |  | 0 (0%) | 0 (0%) | 0 (0%) |  |
| A neighboring county in NY State,Somewhere else in NY State (not a neighboring county) |  | 0 (0%) | 0 (0%) | 0 (0%) |  |
| A neighboring county in NY State,Somewhere else in NY State (not a neighboring county),Another State,International |  | 0 (0%) | 0 (0%) | 0 (0%) |  |
| Another State |  | 9 (17%) | 6 (17%) | 3 (17%) |  |
| I have not traveled outside Tompkins County |  | 23 (53%) | 15 (60%) | 8 (44%) |  |
| International |  | 1 (1.2%) | 0 (0%) | 1 (2.6%) |  |
| Somewhere else in NY State (not a neighboring county) |  | 6 (14%) | 3 (13%) | 3 (14%) |  |
| Somewhere else in NY State (not a neighboring county),Another State |  | 1 (4.1%) | 1 (7.2%) | 0 (0%) |  |
| Travel2 | 0 |  |  |  | 0.252 |
|  |  | 1 (3.9%) | 1 (6.7%) | 0 (0%) |  |
| A neighboring county in NY State |  | 6 (11%) | 1 (2.7%) | 5 (22%) |  |
| Another State |  | 10 (20%) | 7 (23%) | 3 (17%) |  |
| I have not traveled outside Tompkins County |  | 23 (51%) | 15 (56%) | 8 (44%) |  |
| International |  | 1 (1.1%) | 0 (0%) | 1 (2.6%) |  |
| Somewhere else in NY State (not a neighboring county) |  | 6 (13%) | 3 (12%) | 3 (14%) |  |
| Gathering | 2 | 23 (47%) | 14 (51%) | 9 (42%) | 0.662 |
| N\_Gathering | 24 |  |  |  | 0.348 |
| Mean (SE) |  | 2.08 (0.44) | 2.53 (0.80) | 1.41 (0.40) |  |
| Median (IQR) |  | 1.00 (1.00, 2.12) | 1.23 (1.00, 3.87) | 1.00 (1.00, 1.63) |  |
| LargeEvent | 1 | 7 (13%) | 5 (18%) | 2 (7.3%) | 0.267 |
| Gym | 1 |  |  |  | 0.687 |
| 0 |  | 39 (84%) | 21 (81%) | 18 (88%) |  |
| 1 - 3 times |  | 4 (9.3%) | 4 (17%) | 0 (0%) |  |
| 10 - 12 times |  | 0 (0%) | 0 (0%) | 0 (0%) |  |
| 4 - 6 times |  | 2 (5.4%) | 0 (0%) | 2 (12%) |  |
| 7 - 9 times |  | 0 (0%) | 0 (0%) | 0 (0%) |  |
| more than 12 times |  | 1 (1.6%) | 1 (2.9%) | 0 (0%) |  |
| Gym2 | 0 |  |  |  | 0.908 |
|  |  | 1 (3.9%) | 1 (6.7%) | 0 (0%) |  |
| 0 |  | 39 (80%) | 21 (75%) | 18 (88%) |  |
| 1 - 6 times |  | 6 (14%) | 4 (15%) | 2 (12%) |  |
| More than 6 times |  | 1 (1.6%) | 1 (2.7%) | 0 (0%) |  |
| IndoorDining | 1 |  |  |  | 0.777 |
| 0 |  | 28 (63%) | 16 (65%) | 12 (60%) |  |
| 1 - 3 times |  | 15 (32%) | 8 (30%) | 7 (35%) |  |
| 4 - 6 times |  | 1 (2.0%) | 0 (0%) | 1 (4.6%) |  |
| 7 - 9 times |  | 2 (2.5%) | 2 (4.5%) | 0 (0%) |  |
| More than 9 times |  | 0 (0%) | 0 (0%) | 0 (0%) |  |
| IndoorDining2 | 0 |  |  |  | 0.538 |
|  |  | 1 (3.9%) | 1 (6.7%) | 0 (0%) |  |
| 0 |  | 28 (61%) | 16 (61%) | 12 (60%) |  |
| 1 - 3 times |  | 15 (31%) | 8 (28%) | 7 (35%) |  |
| More than 3 times |  | 3 (4.4%) | 2 (4.2%) | 1 (4.6%) |  |
| HandWashing | 1 |  |  |  | 0.785 |
| Decreased this behavior |  | 0 (0%) | 0 (0%) | 0 (0%) |  |
| Haven't changed |  | 11 (21%) | 8 (23%) | 3 (18%) |  |
| Increased this behavior |  | 35 (79%) | 18 (77%) | 17 (82%) |  |
| HandSanitizer | 1 |  |  |  | 0.228 |
| Decreased this behavior |  | 0 (0%) | 0 (0%) | 0 (0%) |  |
| Haven't changed |  | 13 (27%) | 10 (34%) | 3 (17%) |  |
| Increased this behavior |  | 33 (73%) | 16 (66%) | 17 (83%) |  |
| TouchingFace | 1 |  |  |  | 0.567 |
| Decreased this behavior |  | 21 (45%) | 11 (48%) | 10 (42%) |  |
| Haven't changed |  | 22 (48%) | 14 (49%) | 8 (46%) |  |
| Increased this behavior |  | 3 (7.4%) | 1 (3.6%) | 2 (12%) |  |
| Cleaning | 1 |  |  |  | 0.001 |
| Decreased this behavior |  | 0 (0%) | 0 (0%) | 0 (0%) |  |
| Haven't changed |  | 23 (50%) | 18 (72%) | 5 (22%) |  |
| Increased this behavior |  | 23 (50%) | 8 (28%) | 15 (78%) |  |
| StayHomeSick | 1 |  |  |  | 0.597 |
| Decreased this behavior |  | 0 (0%) | 0 (0%) | 0 (0%) |  |
| Haven't changed |  | 19 (40%) | 13 (45%) | 6 (33%) |  |
| Increased this behavior |  | 27 (60%) | 13 (55%) | 14 (67%) |  |
| Doctors | 1 |  |  |  | 0.994 |
| Decreased this behavior |  | 13 (33%) | 6 (29%) | 7 (38%) |  |
| Haven't changed |  | 31 (63%) | 20 (71%) | 11 (53%) |  |
| Increased this behavior |  | 2 (4.1%) | 0 (0%) | 2 (9.2%) |  |
| NursingHome | 1 |  |  |  | 0.750 |
| Decreased this behavior |  | 7 (15%) | 4 (18%) | 3 (11%) |  |
| Haven't changed |  | 37 (80%) | 20 (73%) | 17 (89%) |  |
| Increased this behavior |  | 2 (5.2%) | 2 (9.3%) | 0 (0%) |  |
| Telehealth | 1 |  |  |  | 0.025 |
| Decreased this behavior |  | 1 (2.0%) | 1 (3.6%) | 0 (0%) |  |
| Haven't changed |  | 22 (43%) | 16 (56%) | 6 (26%) |  |
| Increased this behavior |  | 23 (55%) | 9 (40%) | 14 (74%) |  |
| Curbside | 1 |  |  |  | 0.788 |
| Decreased this behavior |  | 0 (0%) | 0 (0%) | 0 (0%) |  |
| Haven't changed |  | 19 (40%) | 11 (42%) | 8 (38%) |  |
| Increased this behavior |  | 27 (60%) | 15 (58%) | 12 (62%) |  |
|  |  |  |  |  |  |
| --- | --- | --- | --- | --- | --- |
| 1 n unweighted (% weighted) | | | | | |
| 2 Wald test of independence for complex survey samples; Wilcoxon rank-sum test for complex survey samples; Kruskal-Wallis rank-sum test for complex survey samples | | | | | |
