# Supplementary material for: Prevalence of SARS-CoV-2 infection and immunity in a New York county in 2022 reveals frequent asymptomatic or undiagnosed infections
Source: PLoS One. 2025 May 28;20(5):e0323659. doi: 10.1371/journal.pone.0323659 (PMC12118914; doi:10.1371/journal.pone.0323659)
Supplement: S11 Table — Table of the univariate comparisons between antibody presence and demographic risk factors for infection in April 2022. (HTML) [file pone.0323659.s011.html]

| **Characteristic** | **N Missing** | **Overall** N=471 | **FALSE** N=271 | **TRUE** N=201 | **p-value**2 |
| --- | --- | --- | --- | --- | --- |
| Age | 0 |  |  |  | 0.456 |
| Mean (SE) |  | 53 (3) | 52 (3) | 54 (5) |  |
| Median (IQR) |  | 55 (42, 63) | 53 (42, 59) | 61 (39, 70) |  |
| Age2 | 0 |  |  |  | 0.100 |
| 18 to 64 |  | 37 (79%) | 23 (90%) | 14 (64%) |  |
| 65 and over |  | 10 (21%) | 4 (10%) | 6 (36%) |  |
| Under 18 |  | 0 (0%) | 0 (0%) | 0 (0%) |  |
| Gender | 0 |  |  |  | 0.271 |
| Female |  | 26 (61%) | 17 (69%) | 9 (50%) |  |
| Gender non-binary |  | 1 (1.6%) | 0 (NA%) | 1 (NA%) |  |
| Male |  | 19 (35%) | 9 (27%) | 10 (46%) |  |
| Other |  | 1 (1.9%) | 1 (3.4%) | 0 (0%) |  |
| Gender2 | 0 |  |  |  | 0.279 |
|  |  | 0 (0%) | 0 (0%) | 0 (0%) |  |
| Female |  | 26 (61%) | 17 (69%) | 9 (50%) |  |
| Male |  | 19 (35%) | 9 (27%) | 10 (46%) |  |
| Other |  | 2 (3.5%) | 1 (3.4%) | 1 (3.7%) |  |
| Race | 1 |  |  |  | 0.359 |
| American Indian or Alaskan Native |  | 0 (0%) | 0 (0%) | 0 (0%) |  |
| American Indian or Alaskan Native,Native Hawaiian or Pacific Islander |  | 0 (0%) | 0 (0%) | 0 (0%) |  |
| American Indian or Alaskan Native,White |  | 0 (0%) | 0 (0%) | 0 (0%) |  |
| Asian |  | 3 (7.4%) | 1 (3.6%) | 2 (12%) |  |
| Asian,White |  | 0 (0%) | 0 (0%) | 0 (0%) |  |
| Black or African American |  | 1 (1.4%) | 0 (0%) | 1 (3.1%) |  |
| White |  | 42 (91%) | 25 (96%) | 17 (85%) |  |
| Race2 | 0 |  |  |  | 0.363 |
|  |  | 1 (3.9%) | 1 (6.7%) | 0 (0%) |  |
| American Indian or Alaskan Native |  | 0 (0%) | 0 (0%) | 0 (0%) |  |
| Asian |  | 3 (7.1%) | 1 (3.4%) | 2 (12%) |  |
| Black or African American |  | 1 (1.3%) | 0 (0%) | 1 (3.1%) |  |
| Two or more races |  | 0 (0%) | 0 (0%) | 0 (0%) |  |
| White |  | 42 (88%) | 25 (90%) | 17 (85%) |  |
| Ethnicity | 2 |  |  |  | 0.759 |
| Hispanic or Latino |  | 2 (3.7%) | 1 (3.0%) | 1 (4.6%) |  |
| Not Hispanic or Latino |  | 43 (96%) | 24 (97%) | 19 (95%) |  |
| Education | 1 |  |  |  | 0.845 |
| Associate's Degree |  | 2 (4.1%) | 1 (3.6%) | 1 (4.6%) |  |
| Bachelor's Degree |  | 4 (11%) | 2 (10%) | 2 (12%) |  |
| Between grades 9 - 11 |  | 0 (0%) | 0 (0%) | 0 (0%) |  |
| Graduate/ Professional School Degree |  | 36 (77%) | 21 (80%) | 15 (72%) |  |
| High School Diploma / GED certificate |  | 4 (8.0%) | 2 (5.8%) | 2 (11%) |  |
| none or between grades 1 - 8 |  | 0 (0%) | 0 (0%) | 0 (0%) |  |
| Technical, trade or vocational school |  | 0 (0%) | 0 (0%) | 0 (0%) |  |
| Education2 | 7 |  |  |  | 0.808 |
| No Bachelor's Degree |  | 0 (0%) | 0 (0%) | 0 (0%) |  |
| Bachelor's Degree |  | 4 (13%) | 2 (11%) | 2 (15%) |  |
| Graduate/ Professional School Degree |  | 36 (87%) | 21 (89%) | 15 (85%) |  |
| HH Size | 1 |  |  |  | 0.277 |
| Mean (SE) |  | 2.54 (0.28) | 2.79 (0.37) | 2.22 (0.37) |  |
| Median (IQR) |  | 2.00 (1.33, 4.00) | 2.00 (2.00, 4.00) | 2.00 (1.00, 3.00) |  |
| Number of Generations in HH | 13 |  |  |  | 0.209 |
| 1 |  | 15 (44%) | 7 (34%) | 8 (59%) |  |
| 2 |  | 18 (53%) | 12 (61%) | 6 (41%) |  |
| 3 |  | 1 (2.9%) | 1 (5.0%) | 0 (0%) |  |
|  |  |  |  |  |  |
| --- | --- | --- | --- | --- | --- |
| 1 n unweighted (% weighted) | | | | | |
| 2 Kruskal-Wallis rank-sum test for complex survey samples; Wald test of independence for complex survey samples | | | | | |
