# Supplementary material for: Prevalence of SARS-CoV-2 infection and immunity in a New York county in 2022 reveals frequent asymptomatic or undiagnosed infections
Source: PLoS One. 2025 May 28;20(5):e0323659. doi: 10.1371/journal.pone.0323659 (PMC12118914; doi:10.1371/journal.pone.0323659)
Supplement: S12 Table — Table of the univariate comparisons between antibody presence and work risk factors for infection in April 2022. (HTML) [file pone.0323659.s012.html]

| **Characteristic** | **N Missing** | **Overall** N=471 | **FALSE** N=271 | **TRUE** N=201 | **p-value**2 |
| --- | --- | --- | --- | --- | --- |
| Employment | 1 |  |  |  | 0.415 |
| Disabled |  | 0 (0%) | 0 (0%) | 0 (0%) |  |
| Full time employment |  | 26 (56%) | 16 (62%) | 10 (47%) |  |
| Full time employment,Self-employed |  | 1 (1.6%) | 0 (0%) | 1 (3.7%) |  |
| Full time employment,Student |  | 0 (0%) | 0 (0%) | 0 (0%) |  |
| Part time employment |  | 6 (14%) | 2 (5.7%) | 4 (24%) |  |
| Part time employment,Self-employed |  | 0 (0%) | 0 (0%) | 0 (0%) |  |
| Part time employment,Student |  | 0 (0%) | 0 (0%) | 0 (0%) |  |
| Retired |  | 7 (13%) | 4 (11%) | 3 (16%) |  |
| Self-employed |  | 4 (9.9%) | 3 (14%) | 1 (4.6%) |  |
| Student |  | 1 (2.0%) | 0 (0%) | 1 (4.6%) |  |
| Unemployed |  | 1 (4.1%) | 1 (7.2%) | 0 (0%) |  |
| Employment2 | 0 |  |  |  | 0.561 |
|  |  | 1 (3.9%) | 1 (6.7%) | 0 (0%) |  |
| Employed |  | 37 (78%) | 21 (76%) | 16 (79%) |  |
| Retired, Unemployed, or Disabled |  | 8 (17%) | 5 (17%) | 3 (16%) |  |
| Student |  | 1 (1.9%) | 0 (0%) | 1 (4.6%) |  |
| WorkLocation | 10 |  |  |  | 0.085 |
| Other (describe): |  | 0 (0%) | 0 (0%) | 0 (0%) |  |
| All remote work |  | 4 (9.9%) | 4 (17%) | 0 (0%) |  |
| Mostly remote (more than half) with some in-person work |  | 7 (23%) | 5 (28%) | 2 (17%) |  |
| About half in-person and half remote |  | 4 (9.0%) | 1 (3.5%) | 3 (16%) |  |
| Mostly in-person (more than half) with some remote work |  | 8 (25%) | 5 (30%) | 3 (19%) |  |
| All in-person (on-site) work |  | 14 (32%) | 6 (21%) | 8 (47%) |  |
| WorkLocation2 | 32 |  |  |  | 0.143 |
| More than half remote |  | 11 (79%) | 9 (93%) | 2 (52%) |  |
| About half in-person and half remote |  | 4 (21%) | 1 (7.2%) | 3 (48%) |  |
| More than half in person |  | 0 (0%) | 0 (0%) | 0 (0%) |  |
| WorkNumContact | 14 |  |  |  | 0.328 |
| 0 to 5 |  | 15 (52%) | 9 (60%) | 6 (43%) |  |
| 6 to 10 |  | 6 (20%) | 2 (14%) | 4 (26%) |  |
| 11 to 20 |  | 6 (13%) | 4 (17%) | 2 (9.7%) |  |
| More than 20 |  | 6 (15%) | 2 (8.9%) | 4 (21%) |  |
| WorkNumContact2 | 14 |  |  |  | 0.742 |
| 0 to 10 |  | 21 (72%) | 11 (74%) | 10 (69%) |  |
| More than 10 |  | 12 (28%) | 6 (26%) | 6 (31%) |  |
| WorkPublic | 14 | 15 (42%) | 7 (35%) | 8 (49%) | 0.395 |
|  |  |  |  |  |  |
| --- | --- | --- | --- | --- | --- |
| 1 n unweighted (% weighted) | | | | | |
| 2 Wald test of independence for complex survey samples; Wilcoxon rank-sum test for complex survey samples | | | | | |
