# Supplementary material for: Prevalence of SARS-CoV-2 infection and immunity in a New York county in 2022 reveals frequent asymptomatic or undiagnosed infections
Source: PLoS One. 2025 May 28;20(5):e0323659. doi: 10.1371/journal.pone.0323659 (PMC12118914; doi:10.1371/journal.pone.0323659)
Supplement: S13 Table — Table of the univariate comparisons between antibody presence and attitude risk factors for infection in February 2022. (HTML) [file pone.0323659.s013.html]

| **Characteristic** | **N Missing** | **Overall** N=861 | **FALSE** N=491 | **TRUE** N=371 | **p-value**2 |
| --- | --- | --- | --- | --- | --- |
| DistanceImportant | 1 |  |  |  | 0.815 |
| Strongly agree |  | 37 (47%) | 21 (45%) | 16 (50%) |  |
| Agree |  | 26 (24%) | 17 (25%) | 9 (23%) |  |
| Neither agree nor disagree |  | 6 (9.4%) | 3 (12%) | 3 (6.2%) |  |
| Disagree |  | 3 (4.9%) | 1 (3.5%) | 2 (6.8%) |  |
| Strongly disagree |  | 13 (14%) | 6 (14%) | 7 (14%) |  |
| MaskImportant | 1 |  |  |  | 0.754 |
| Strongly agree |  | 48 (61%) | 27 (63%) | 21 (59%) |  |
| Agree |  | 19 (19%) | 13 (19%) | 6 (19%) |  |
| Neither agree nor disagree |  | 4 (3.6%) | 2 (4.1%) | 2 (3.0%) |  |
| Disagree |  | 1 (1.8%) | 0 (0%) | 1 (4.2%) |  |
| Strongly disagree |  | 13 (14%) | 6 (14%) | 7 (14%) |  |
| TravelImportant | 1 |  |  |  | 0.085 |
| Strongly agree |  | 21 (26%) | 10 (17%) | 11 (37%) |  |
| Agree |  | 40 (46%) | 23 (51%) | 17 (40%) |  |
| Neither agree nor disagree |  | 14 (15%) | 9 (19%) | 5 (10%) |  |
| Disagree |  | 7 (7.1%) | 4 (5.7%) | 3 (8.9%) |  |
| Strongly disagree |  | 3 (5.7%) | 2 (7.5%) | 1 (3.5%) |  |
| Worried | 1 |  |  |  | 0.148 |
| Not at all worried |  | 15 (15%) | 6 (9.7%) | 9 (21%) |  |
| Not that worried |  | 36 (41%) | 21 (38%) | 15 (44%) |  |
| Somewhat worried |  | 26 (34%) | 16 (43%) | 10 (23%) |  |
| Very worried |  | 8 (10%) | 5 (9.4%) | 3 (12%) |  |
| FollowProtocols | 1 |  |  |  | 0.527 |
| Strongly agree |  | 39 (48%) | 21 (45%) | 18 (53%) |  |
| Agree |  | 32 (36%) | 18 (38%) | 14 (34%) |  |
| Neither agree nor disagree |  | 7 (8.7%) | 4 (9.4%) | 3 (7.9%) |  |
| Disagree |  | 4 (4.6%) | 2 (4.1%) | 2 (5.2%) |  |
| Strongly disagree |  | 3 (2.4%) | 3 (4.3%) | 0 (0%) |  |
| DistanceImportant2 | 1 |  |  |  | 0.964 |
| Agree |  | 63 (72%) | 38 (71%) | 25 (73%) |  |
| Neither agree nor disagree |  | 6 (9.4%) | 3 (12%) | 3 (6.2%) |  |
| Disagree |  | 16 (19%) | 7 (17%) | 9 (21%) |  |
| MaskImportant2 | 1 |  |  |  | 0.692 |
| Agree |  | 67 (81%) | 40 (82%) | 27 (79%) |  |
| Neither agree nor disagree |  | 4 (3.6%) | 2 (4.1%) | 2 (3.0%) |  |
| Disagree |  | 14 (16%) | 6 (14%) | 8 (18%) |  |
| TravelImportant2 | 1 |  |  |  | 0.484 |
| Agree |  | 61 (72%) | 33 (68%) | 28 (77%) |  |
| Neither agree nor disagree |  | 14 (15%) | 9 (19%) | 5 (10%) |  |
| Disagree |  | 10 (13%) | 6 (13%) | 4 (12%) |  |
| FollowProtocols2 | 1 |  |  |  | 0.622 |
| Agree |  | 71 (84%) | 39 (82%) | 32 (87%) |  |
| Neither agree nor disagree |  | 7 (8.7%) | 4 (9.4%) | 3 (7.9%) |  |
| Disagree |  | 7 (7.0%) | 5 (8.3%) | 2 (5.2%) |  |
|  |  |  |  |  |  |
| --- | --- | --- | --- | --- | --- |
| 1 n unweighted (% weighted) | | | | | |
| 2 Wilcoxon rank-sum test for complex survey samples | | | | | |
