# Supplementary material for: Prevalence of SARS-CoV-2 infection and immunity in a New York county in 2022 reveals frequent asymptomatic or undiagnosed infections
Source: PLoS One. 2025 May 28;20(5):e0323659. doi: 10.1371/journal.pone.0323659 (PMC12118914; doi:10.1371/journal.pone.0323659)
Supplement: S14 Table — Table of the univariate comparisons between antibody presence and behavior risk factors for infection in February 2022. (HTML) [file pone.0323659.s014.html]

| **Characteristic** | **N Missing** | **Overall** N=861 | **FALSE** N=491 | **TRUE** N=371 | **p-value**2 |
| --- | --- | --- | --- | --- | --- |
| Smoking | 1 |  |  |  | 0.692 |
| current smoker |  | 9 (8.9%) | 6 (11%) | 3 (6.2%) |  |
| former smoker |  | 14 (16%) | 9 (18%) | 5 (14%) |  |
| never smoked |  | 62 (75%) | 33 (71%) | 29 (80%) |  |
| Smoking2 | 0 |  |  |  | 0.486 |
|  |  | 1 (1.1%) | 1 (2.0%) | 0 (0%) |  |
| current or former smoker |  | 23 (25%) | 15 (28%) | 8 (20%) |  |
| never smoked |  | 62 (74%) | 33 (70%) | 29 (80%) |  |
| Vaping | 1 |  |  |  | 0.505 |
| current user |  | 3 (3.7%) | 1 (1.6%) | 2 (6.3%) |  |
| former user |  | 7 (8.2%) | 4 (10%) | 3 (5.4%) |  |
| never used a vaping device |  | 75 (88%) | 43 (88%) | 32 (88%) |  |
| Vaping2 | 0 |  |  |  | 0.635 |
|  |  | 1 (1.1%) | 1 (2.0%) | 0 (0%) |  |
| current or former vaper |  | 10 (12%) | 5 (12%) | 5 (12%) |  |
| never used a vaping device |  | 75 (87%) | 43 (86%) | 32 (88%) |  |
| Masking | 1 |  |  |  | 0.698 |
| I don't go to public places |  | 0 (0%) | 0 (0%) | 0 (0%) |  |
| Always |  | 71 (85%) | 41 (87%) | 30 (82%) |  |
| More than half of the time |  | 9 (11%) | 5 (9.6%) | 4 (12%) |  |
| About half of the time |  | 4 (3.5%) | 2 (3.7%) | 2 (3.2%) |  |
| Less than half of the time |  | 1 (1.1%) | 0 (0%) | 1 (2.6%) |  |
| Never |  | 0 (0%) | 0 (0%) | 0 (0%) |  |
| Masking2 | 1 |  |  |  | 0.623 |
| Less than half of the time |  | 1 (1.1%) | 0 (0%) | 1 (2.6%) |  |
| About half of the time |  | 4 (3.5%) | 2 (3.7%) | 2 (3.2%) |  |
| More than half of the time |  | 80 (95%) | 46 (96%) | 34 (94%) |  |
| MaskType | 1 |  |  |  | 0.132 |
| Cloth |  | 4 (3.5%) | 4 (6.3%) | 0 (0%) |  |
| Cloth,Other (describe in box): |  | 0 (0%) | 0 (0%) | 0 (0%) |  |
| Cloth,Respirator (such as N95, KN95, FFP2, KF94) |  | 3 (3.0%) | 2 (3.3%) | 1 (2.6%) |  |
| Cloth,Surgical/Medical |  | 8 (7.7%) | 6 (9.4%) | 2 (5.5%) |  |
| Cloth,Surgical/Medical,Other (describe in box): |  | 1 (2.3%) | 1 (4.1%) | 0 (0%) |  |
| Cloth,Surgical/Medical,Respirator (such as N95, KN95, FFP2, KF94) |  | 8 (7.0%) | 5 (6.8%) | 3 (7.3%) |  |
| I never wear a mask |  | 0 (0%) | 0 (0%) | 0 (0%) |  |
| Respirator (such as N95, KN95, FFP2, KF94) |  | 28 (36%) | 15 (34%) | 13 (39%) |  |
| Surgical/Medical |  | 21 (26%) | 9 (19%) | 12 (35%) |  |
| Surgical/Medical,Other (describe in box): |  | 1 (1.1%) | 1 (2.0%) | 0 (0%) |  |
| Surgical/Medical,Respirator (such as N95, KN95, FFP2, KF94) |  | 11 (14%) | 5 (15%) | 6 (11%) |  |
| MaskType2 | 1 |  |  |  | 0.841 |
| I never wear a mask |  | 0 (0%) | 0 (0%) | 0 (0%) |  |
| Cloth |  | 4 (3.5%) | 4 (6.3%) | 0 (0%) |  |
| Surgical/Medical |  | 31 (37%) | 17 (34%) | 14 (40%) |  |
| Respirator |  | 50 (60%) | 27 (59%) | 23 (60%) |  |
| Distancing | 1 |  |  |  | 0.634 |
| I don't go to public places |  | 1 (1.5%) | 0 (0%) | 1 (3.5%) |  |
| Always |  | 18 (26%) | 11 (25%) | 7 (26%) |  |
| More than half of the time |  | 29 (30%) | 16 (28%) | 13 (33%) |  |
| About half of the time |  | 13 (15%) | 10 (22%) | 3 (5.3%) |  |
| Less than half of the time |  | 14 (15%) | 5 (9.4%) | 9 (21%) |  |
| Never |  | 10 (13%) | 6 (15%) | 4 (11%) |  |
| Distancing2 | 1 |  |  |  | 0.694 |
| Less than half of the time |  | 24 (28%) | 11 (25%) | 13 (32%) |  |
| About half of the time |  | 13 (15%) | 10 (22%) | 3 (5.3%) |  |
| More than half of the time |  | 48 (57%) | 27 (53%) | 21 (63%) |  |
| Bus | 2 |  |  |  | 0.684 |
| not at all |  | 62 (76%) | 36 (73%) | 26 (80%) |  |
| once |  | 4 (6.7%) | 3 (11%) | 1 (1.3%) |  |
| twice |  | 4 (4.6%) | 3 (6.5%) | 1 (2.2%) |  |
| 3-5 times |  | 8 (6.9%) | 4 (5.7%) | 4 (8.5%) |  |
| 6-10 times |  | 4 (3.3%) | 1 (1.6%) | 3 (5.6%) |  |
| more than 10 times |  | 2 (2.1%) | 1 (2.0%) | 1 (2.2%) |  |
| Bus2 | 2 |  |  |  | 0.602 |
| not at all |  | 62 (76%) | 36 (73%) | 26 (80%) |  |
| 1-5 times |  | 16 (18%) | 10 (23%) | 6 (12%) |  |
| More than 5 times |  | 6 (5.4%) | 2 (3.7%) | 4 (7.7%) |  |
| Plane | 3 |  |  |  | 0.915 |
| not at all |  | 72 (87%) | 42 (86%) | 30 (88%) |  |
| once |  | 6 (8.4%) | 3 (8.7%) | 3 (8.1%) |  |
| twice |  | 2 (1.9%) | 2 (3.3%) | 0 (0%) |  |
| 3-5 times |  | 3 (2.8%) | 1 (1.6%) | 2 (4.4%) |  |
| 6-10 times |  | 0 (0%) | 0 (0%) | 0 (0%) |  |
| more than 10 times |  | 0 (0%) | 0 (0%) | 0 (0%) |  |
| Plane2 | 3 |  |  |  | 0.902 |
| not at all |  | 72 (87%) | 42 (86%) | 30 (88%) |  |
| 1-5 times |  | 11 (13%) | 6 (14%) | 5 (12%) |  |
| More than 5 times |  | 0 (0%) | 0 (0%) | 0 (0%) |  |
| Train | 4 |  |  |  | 0.977 |
| not at all |  | 80 (98%) | 46 (98%) | 34 (98%) |  |
| once |  | 1 (1.0%) | 1 (1.7%) | 0 (0%) |  |
| twice |  | 1 (0.7%) | 0 (0%) | 1 (1.6%) |  |
| 3-5 times |  | 0 (0%) | 0 (0%) | 0 (0%) |  |
| 6-10 times |  | 0 (0%) | 0 (0%) | 0 (0%) |  |
| more than 10 times |  | 0 (0%) | 0 (0%) | 0 (0%) |  |
| Train2 | 4 |  |  |  | 0.968 |
| not at all |  | 80 (98%) | 46 (98%) | 34 (98%) |  |
| 1-5 times |  | 2 (1.7%) | 1 (1.7%) | 1 (1.6%) |  |
| More than 5 times |  | 0 (0%) | 0 (0%) | 0 (0%) |  |
| Metro | 3 |  |  |  | 0.015 |
| not at all |  | 76 (93%) | 47 (98%) | 29 (85%) |  |
| once |  | 0 (0%) | 0 (0%) | 0 (0%) |  |
| twice |  | 3 (3.3%) | 0 (0%) | 3 (7.8%) |  |
| 3-5 times |  | 3 (2.3%) | 1 (1.6%) | 2 (3.2%) |  |
| 6-10 times |  | 1 (1.9%) | 0 (0%) | 1 (4.5%) |  |
| more than 10 times |  | 0 (0%) | 0 (0%) | 0 (0%) |  |
| Metro2 | 3 |  |  |  | 0.015 |
| not at all |  | 76 (93%) | 47 (98%) | 29 (85%) |  |
| 1-5 times |  | 6 (5.6%) | 1 (1.6%) | 5 (11%) |  |
| More than 5 times |  | 1 (1.9%) | 0 (0%) | 1 (4.5%) |  |
| Cab | 3 |  |  |  | 0.735 |
| not at all |  | 62 (76%) | 37 (78%) | 25 (73%) |  |
| once |  | 5 (6.1%) | 3 (5.3%) | 2 (7.2%) |  |
| twice |  | 8 (10%) | 3 (9.1%) | 5 (12%) |  |
| 3-5 times |  | 6 (5.9%) | 3 (4.3%) | 3 (8.1%) |  |
| 6-10 times |  | 0 (0%) | 0 (0%) | 0 (0%) |  |
| more than 10 times |  | 2 (2.1%) | 2 (3.7%) | 0 (0%) |  |
| Cab2 | 3 |  |  |  | 0.751 |
| not at all |  | 62 (76%) | 37 (78%) | 25 (73%) |  |
| 1-5 times |  | 19 (22%) | 9 (19%) | 10 (27%) |  |
| More than 5 times |  | 2 (2.1%) | 2 (3.7%) | 0 (0%) |  |
| Travel | 1 |  |  |  | 0.218 |
| A neighboring county in NY State |  | 13 (16%) | 6 (10%) | 7 (23%) |  |
| A neighboring county in NY State,Another State |  | 0 (0%) | 0 (0%) | 0 (0%) |  |
| A neighboring county in NY State,Somewhere else in NY State (not a neighboring county) |  | 2 (2.3%) | 2 (4.1%) | 0 (0%) |  |
| A neighboring county in NY State,Somewhere else in NY State (not a neighboring county),Another State,International |  | 1 (0.9%) | 1 (1.6%) | 0 (0%) |  |
| Another State |  | 9 (8.0%) | 7 (9.9%) | 2 (5.6%) |  |
| I have not traveled outside Tompkins County |  | 53 (65%) | 28 (66%) | 25 (64%) |  |
| International |  | 3 (3.2%) | 1 (1.6%) | 2 (5.2%) |  |
| Somewhere else in NY State (not a neighboring county) |  | 3 (2.5%) | 2 (3.2%) | 1 (1.5%) |  |
| Somewhere else in NY State (not a neighboring county),Another State |  | 1 (2.0%) | 1 (3.5%) | 0 (0%) |  |
| Travel2 | 0 |  |  |  | 0.149 |
|  |  | 1 (1.1%) | 1 (2.0%) | 0 (0%) |  |
| A neighboring county in NY State |  | 13 (16%) | 6 (10%) | 7 (23%) |  |
| Another State |  | 10 (9.9%) | 8 (13%) | 2 (5.6%) |  |
| I have not traveled outside Tompkins County |  | 53 (64%) | 28 (64%) | 25 (64%) |  |
| International |  | 4 (4.1%) | 2 (3.2%) | 2 (5.2%) |  |
| Somewhere else in NY State (not a neighboring county) |  | 5 (4.7%) | 4 (7.1%) | 1 (1.5%) |  |
| Gathering | 1 | 52 (62%) | 28 (63%) | 24 (60%) | 0.733 |
| N\_Gathering | 34 |  |  |  | 0.683 |
| Mean (SE) |  | 3.84 (0.56) | 4.22 (0.88) | 3.33 (0.45) |  |
| Median (IQR) |  | 3.00 (2.00, 4.00) | 3.00 (2.00, 5.00) | 3.00 (2.00, 4.00) |  |
| LargeEvent | 1 | 4 (4.4%) | 4 (7.8%) | 0 (0%) | 0.086 |
| Gym | 1 |  |  |  | 0.357 |
| 0 |  | 58 (67%) | 36 (74%) | 22 (59%) |  |
| 1 - 3 times |  | 11 (9.3%) | 5 (7.3%) | 6 (12%) |  |
| 10 - 12 times |  | 3 (3.0%) | 2 (2.6%) | 1 (3.5%) |  |
| 4 - 6 times |  | 7 (9.3%) | 2 (3.7%) | 5 (17%) |  |
| 7 - 9 times |  | 3 (2.1%) | 1 (1.0%) | 2 (3.6%) |  |
| more than 12 times |  | 3 (8.8%) | 2 (12%) | 1 (5.2%) |  |
| Gym2 | 0 |  |  |  | 0.353 |
|  |  | 1 (1.1%) | 1 (2.0%) | 0 (0%) |  |
| 0 |  | 58 (67%) | 36 (72%) | 22 (59%) |  |
| 1 - 6 times |  | 18 (18%) | 7 (11%) | 11 (28%) |  |
| More than 6 times |  | 9 (14%) | 5 (15%) | 4 (12%) |  |
| IndoorDining | 1 |  |  |  | 0.175 |
| 0 |  | 37 (40%) | 26 (47%) | 11 (32%) |  |
| 1 - 3 times |  | 39 (50%) | 18 (46%) | 21 (55%) |  |
| 4 - 6 times |  | 6 (7.3%) | 3 (6.1%) | 3 (8.9%) |  |
| 7 - 9 times |  | 0 (0%) | 0 (0%) | 0 (0%) |  |
| More than 9 times |  | 3 (2.7%) | 1 (1.6%) | 2 (4.1%) |  |
| IndoorDining2 | 0 |  |  |  | 0.130 |
|  |  | 1 (1.1%) | 1 (2.0%) | 0 (0%) |  |
| 0 |  | 37 (40%) | 26 (46%) | 11 (32%) |  |
| 1 - 3 times |  | 39 (49%) | 18 (45%) | 21 (55%) |  |
| More than 3 times |  | 9 (9.9%) | 4 (7.6%) | 5 (13%) |  |
| HandWashing | 1 |  |  |  | 0.548 |
| Decreased this behavior |  | 1 (1.1%) | 0 (0%) | 1 (2.6%) |  |
| Haven't changed |  | 20 (25%) | 9 (23%) | 11 (28%) |  |
| Increased this behavior |  | 64 (74%) | 39 (77%) | 25 (69%) |  |
| HandSanitizer | 2 |  |  |  | 0.434 |
| Decreased this behavior |  | 2 (2.3%) | 0 (0%) | 2 (5.2%) |  |
| Haven't changed |  | 11 (12%) | 6 (11%) | 5 (13%) |  |
| Increased this behavior |  | 71 (86%) | 41 (89%) | 30 (82%) |  |
| TouchingFace | 1 |  |  |  | 0.968 |
| Decreased this behavior |  | 37 (42%) | 21 (41%) | 16 (44%) |  |
| Haven't changed |  | 45 (54%) | 26 (58%) | 19 (49%) |  |
| Increased this behavior |  | 3 (4.4%) | 1 (1.6%) | 2 (7.9%) |  |
| Cleaning | 2 |  |  |  | 0.284 |
| Decreased this behavior |  | 2 (2.3%) | 0 (0%) | 2 (5.3%) |  |
| Haven't changed |  | 35 (44%) | 19 (42%) | 16 (46%) |  |
| Increased this behavior |  | 47 (54%) | 29 (58%) | 18 (48%) |  |
| StayHomeSick | 1 |  |  |  | 0.427 |
| Decreased this behavior |  | 1 (1.1%) | 0 (0%) | 1 (2.6%) |  |
| Haven't changed |  | 25 (26%) | 14 (24%) | 11 (29%) |  |
| Increased this behavior |  | 59 (73%) | 34 (76%) | 25 (69%) |  |
| Doctors | 1 |  |  |  | 0.346 |
| Decreased this behavior |  | 25 (26%) | 16 (25%) | 9 (27%) |  |
| Haven't changed |  | 54 (68%) | 26 (63%) | 28 (73%) |  |
| Increased this behavior |  | 6 (6.4%) | 6 (11%) | 0 (0%) |  |
| NursingHome | 2 |  |  |  | 0.251 |
| Decreased this behavior |  | 12 (13%) | 5 (8.9%) | 7 (19%) |  |
| Haven't changed |  | 72 (87%) | 42 (91%) | 30 (81%) |  |
| Increased this behavior |  | 0 (0%) | 0 (0%) | 0 (0%) |  |
| Telehealth | 2 |  |  |  | 0.006 |
| Decreased this behavior |  | 1 (0.6%) | 1 (1.0%) | 0 (0%) |  |
| Haven't changed |  | 34 (41%) | 13 (26%) | 21 (60%) |  |
| Increased this behavior |  | 49 (59%) | 33 (73%) | 16 (40%) |  |
| Curbside | 1 |  |  |  | 0.844 |
| Decreased this behavior |  | 3 (3.1%) | 2 (3.4%) | 1 (2.6%) |  |
| Haven't changed |  | 17 (21%) | 10 (22%) | 7 (21%) |  |
| Increased this behavior |  | 65 (76%) | 36 (75%) | 29 (77%) |  |
|  |  |  |  |  |  |
| --- | --- | --- | --- | --- | --- |
| 1 n unweighted (% weighted) | | | | | |
| 2 Wald test of independence for complex survey samples; Wilcoxon rank-sum test for complex survey samples; Kruskal-Wallis rank-sum test for complex survey samples | | | | | |
