# Supplementary material for: Prevalence of SARS-CoV-2 infection and immunity in a New York county in 2022 reveals frequent asymptomatic or undiagnosed infections
Source: PLoS One. 2025 May 28;20(5):e0323659. doi: 10.1371/journal.pone.0323659 (PMC12118914; doi:10.1371/journal.pone.0323659)
Supplement: S15 Table — Table of the univariate comparisons between antibody presence and demographic risk factors for infection in February 2022. (HTML) [file pone.0323659.s015.html]

| **Characteristic** | **N Missing** | **Overall** N=861 | **FALSE** N=491 | **TRUE** N=371 | **p-value**2 |
| --- | --- | --- | --- | --- | --- |
| Age | 0 |  |  |  | 0.063 |
| Mean (SE) |  | 38 (3) | 42 (3) | 33 (3) |  |
| Median (IQR) |  | 29 (23, 50) | 32 (25, 56) | 27 (22, 33) |  |
| Age2 | 0 |  |  |  | 0.152 |
| 18 to 64 |  | 74 (83%) | 40 (77%) | 34 (91%) |  |
| 65 and over |  | 12 (17%) | 9 (23%) | 3 (8.7%) |  |
| Under 18 |  | 0 (0%) | 0 (0%) | 0 (0%) |  |
| Gender | 0 |  |  |  | 0.455 |
| Female |  | 47 (53%) | 30 (59%) | 17 (44%) |  |
| Gender non-binary |  | 0 (0%) | 0 (NA%) | 0 (NA%) |  |
| Male |  | 35 (43%) | 17 (36%) | 18 (52%) |  |
| Other |  | 4 (4.6%) | 2 (5.0%) | 2 (4.1%) |  |
| Gender2 | 0 |  |  |  | 0.455 |
|  |  | 0 (0%) | 0 (0%) | 0 (0%) |  |
| Female |  | 47 (53%) | 30 (59%) | 17 (44%) |  |
| Male |  | 35 (43%) | 17 (36%) | 18 (52%) |  |
| Other |  | 4 (4.6%) | 2 (5.0%) | 2 (4.1%) |  |
| Race | 1 |  |  |  | 0.285 |
| American Indian or Alaskan Native |  | 0 (0%) | 0 (0%) | 0 (0%) |  |
| American Indian or Alaskan Native,Native Hawaiian or Pacific Islander |  | 1 (1.5%) | 0 (0%) | 1 (3.5%) |  |
| American Indian or Alaskan Native,White |  | 1 (1.8%) | 0 (0%) | 1 (4.2%) |  |
| Asian |  | 18 (16%) | 10 (14%) | 8 (19%) |  |
| Asian,White |  | 1 (2.3%) | 0 (0%) | 1 (5.3%) |  |
| Black or African American |  | 2 (1.7%) | 2 (3.0%) | 0 (0%) |  |
| White |  | 62 (76%) | 37 (83%) | 25 (68%) |  |
| Race2 | 0 |  |  |  | 0.179 |
|  |  | 1 (0.6%) | 0 (0%) | 1 (1.3%) |  |
| American Indian or Alaskan Native |  | 0 (0%) | 0 (0%) | 0 (0%) |  |
| Asian |  | 18 (16%) | 10 (14%) | 8 (19%) |  |
| Black or African American |  | 2 (1.7%) | 2 (3.0%) | 0 (0%) |  |
| Two or more races |  | 3 (5.6%) | 0 (0%) | 3 (13%) |  |
| White |  | 62 (76%) | 37 (83%) | 25 (67%) |  |
| Ethnicity | 2 |  |  |  | 0.360 |
| Hispanic or Latino |  | 3 (3.0%) | 1 (1.6%) | 2 (5.0%) |  |
| Not Hispanic or Latino |  | 81 (97%) | 48 (98%) | 33 (95%) |  |
| Education | 0 |  |  |  | 0.325 |
| Associate's Degree |  | 2 (1.7%) | 2 (2.9%) | 0 (0%) |  |
| Bachelor's Degree |  | 30 (33%) | 15 (29%) | 15 (39%) |  |
| Between grades 9 - 11 |  | 0 (0%) | 0 (0%) | 0 (0%) |  |
| Graduate/ Professional School Degree |  | 41 (48%) | 23 (48%) | 18 (50%) |  |
| High School Diploma / GED certificate |  | 12 (16%) | 8 (19%) | 4 (11%) |  |
| none or between grades 1 - 8 |  | 1 (1.1%) | 1 (2.0%) | 0 (0%) |  |
| Technical, trade or vocational school |  | 0 (0%) | 0 (0%) | 0 (0%) |  |
| Education2 | 15 |  |  |  | 0.681 |
| No Bachelor's Degree |  | 0 (0%) | 0 (0%) | 0 (0%) |  |
| Bachelor's Degree |  | 30 (41%) | 15 (38%) | 15 (44%) |  |
| Graduate/ Professional School Degree |  | 41 (59%) | 23 (62%) | 18 (56%) |  |
| HH Size | 1 |  |  |  | 0.077 |
| Mean (SE) |  | 3.07 (0.36) | 2.68 (0.34) | 3.58 (0.62) |  |
| Median (IQR) |  | 2.00 (2.00, 4.00) | 2.00 (2.00, 3.00) | 3.00 (2.00, 4.00) |  |
| Number of Generations in HH | 24 |  |  |  | 0.529 |
| 1 |  | 49 (80%) | 28 (86%) | 21 (72%) |  |
| 2 |  | 11 (17%) | 5 (11%) | 6 (25%) |  |
| 3 |  | 2 (3.1%) | 1 (2.7%) | 1 (3.5%) |  |
|  |  |  |  |  |  |
| --- | --- | --- | --- | --- | --- |
| 1 n unweighted (% weighted) | | | | | |
| 2 Kruskal-Wallis rank-sum test for complex survey samples; Wald test of independence for complex survey samples | | | | | |
