# Supplementary material for: Prevalence of SARS-CoV-2 infection and immunity in a New York county in 2022 reveals frequent asymptomatic or undiagnosed infections
Source: PLoS One. 2025 May 28;20(5):e0323659. doi: 10.1371/journal.pone.0323659 (PMC12118914; doi:10.1371/journal.pone.0323659)
Supplement: S16 Table — Table of the univariate comparisons between antibody presence and work risk factors for infection in February 2022. (HTML) [file pone.0323659.s016.html]

| **Characteristic** | **N Missing** | **Overall** N=861 | **FALSE** N=491 | **TRUE** N=371 | **p-value**2 |
| --- | --- | --- | --- | --- | --- |
| Employment | 0 |  |  |  | 0.349 |
| Disabled |  | 0 (0%) | 0 (0%) | 0 (0%) |  |
| Full time employment |  | 23 (24%) | 14 (23%) | 9 (26%) |  |
| Full time employment,Self-employed |  | 0 (0%) | 0 (0%) | 0 (0%) |  |
| Full time employment,Student |  | 5 (5.5%) | 3 (6.6%) | 2 (4.2%) |  |
| Part time employment |  | 4 (4.9%) | 2 (3.3%) | 2 (7.0%) |  |
| Part time employment,Self-employed |  | 0 (0%) | 0 (0%) | 0 (0%) |  |
| Part time employment,Student |  | 1 (0.9%) | 1 (1.6%) | 0 (0%) |  |
| Retired |  | 11 (16%) | 9 (23%) | 2 (6.1%) |  |
| Self-employed |  | 4 (4.0%) | 2 (3.0%) | 2 (5.2%) |  |
| Student |  | 31 (38%) | 15 (35%) | 16 (42%) |  |
| Unemployed |  | 7 (7.0%) | 3 (5.0%) | 4 (9.5%) |  |
| Employment2 | 0 |  |  |  | 0.456 |
|  |  | 0 (0%) | 0 (0%) | 0 (0%) |  |
| Employed |  | 31 (33%) | 18 (29%) | 13 (38%) |  |
| Retired, Unemployed, or Disabled |  | 18 (23%) | 12 (28%) | 6 (16%) |  |
| Student |  | 37 (45%) | 19 (43%) | 18 (46%) |  |
| WorkLocation | 49 |  |  |  | 0.865 |
| Other (describe): |  | 1 (1.9%) | 1 (3.6%) | 0 (0%) |  |
| All remote work |  | 7 (22%) | 4 (20%) | 3 (25%) |  |
| Mostly remote (more than half) with some in-person work |  | 4 (11%) | 3 (11%) | 1 (10.0%) |  |
| About half in-person and half remote |  | 6 (18%) | 3 (19%) | 3 (16%) |  |
| Mostly in-person (more than half) with some remote work |  | 12 (28%) | 7 (28%) | 5 (27%) |  |
| All in-person (on-site) work |  | 7 (19%) | 4 (18%) | 3 (22%) |  |
| WorkLocation2 | 69 |  |  |  | 0.815 |
| More than half remote |  | 11 (65%) | 7 (63%) | 4 (68%) |  |
| About half in-person and half remote |  | 6 (35%) | 3 (37%) | 3 (32%) |  |
| More than half in person |  | 0 (0%) | 0 (0%) | 0 (0%) |  |
| WorkNumContact | 57 |  |  |  | 0.947 |
| 0 to 5 |  | 14 (47%) | 7 (43%) | 7 (52%) |  |
| 6 to 10 |  | 3 (13%) | 2 (19%) | 1 (6.6%) |  |
| 11 to 20 |  | 4 (13%) | 3 (15%) | 1 (11%) |  |
| More than 20 |  | 8 (27%) | 5 (23%) | 3 (30%) |  |
| WorkNumContact2 | 57 |  |  |  | 0.895 |
| 0 to 10 |  | 17 (60%) | 9 (62%) | 8 (59%) |  |
| More than 10 |  | 12 (40%) | 8 (38%) | 4 (41%) |  |
| WorkPublic | 57 | 10 (30%) | 8 (37%) | 2 (22%) | 0.396 |
|  |  |  |  |  |  |
| --- | --- | --- | --- | --- | --- |
| 1 n unweighted (% weighted) | | | | | |
| 2 Wald test of independence for complex survey samples; Wilcoxon rank-sum test for complex survey samples | | | | | |
