# Supplementary material for: Prevalence of SARS-CoV-2 infection and immunity in a New York county in 2022 reveals frequent asymptomatic or undiagnosed infections
Source: PLoS One. 2025 May 28;20(5):e0323659. doi: 10.1371/journal.pone.0323659 (PMC12118914; doi:10.1371/journal.pone.0323659)
Supplement: S17 Table — Table of the univariate comparisons between antibody presence and attitude risk factors for infection in October 2022. (HTML) [file pone.0323659.s017.html]

| **Characteristic** | **N Missing** | **Overall** N=471 | **FALSE** N=241 | **TRUE** N=231 | **p-value**2 |
| --- | --- | --- | --- | --- | --- |
| DistanceImportant | 0 |  |  |  | 0.904 |
| Strongly agree |  | 17 (36%) | 11 (41%) | 6 (30%) |  |
| Agree |  | 20 (43%) | 6 (28%) | 14 (58%) |  |
| Neither agree nor disagree |  | 3 (4.9%) | 2 (7.1%) | 1 (2.4%) |  |
| Disagree |  | 0 (0%) | 0 (0%) | 0 (0%) |  |
| Strongly disagree |  | 7 (17%) | 5 (24%) | 2 (9.6%) |  |
| MaskImportant | 0 |  |  |  | 0.918 |
| Strongly agree |  | 21 (46%) | 12 (52%) | 9 (41%) |  |
| Agree |  | 16 (32%) | 5 (21%) | 11 (45%) |  |
| Neither agree nor disagree |  | 5 (11%) | 3 (12%) | 2 (9.6%) |  |
| Disagree |  | 0 (0%) | 0 (0%) | 0 (0%) |  |
| Strongly disagree |  | 5 (11%) | 4 (16%) | 1 (4.8%) |  |
| TravelImportant | 1 |  |  |  | 0.576 |
| Strongly agree |  | 8 (25%) | 6 (33%) | 2 (17%) |  |
| Agree |  | 21 (42%) | 9 (33%) | 12 (52%) |  |
| Neither agree nor disagree |  | 10 (19%) | 6 (23%) | 4 (14%) |  |
| Disagree |  | 3 (6.6%) | 1 (3.3%) | 2 (10%) |  |
| Strongly disagree |  | 4 (7.1%) | 2 (7.1%) | 2 (7.1%) |  |
| Worried | 1 |  |  |  | 0.348 |
| Not at all worried |  | 9 (21%) | 5 (18%) | 4 (25%) |  |
| Not that worried |  | 19 (39%) | 12 (54%) | 7 (21%) |  |
| Somewhat worried |  | 17 (36%) | 7 (28%) | 10 (46%) |  |
| Very worried |  | 1 (3.6%) | 0 (0%) | 1 (7.8%) |  |
| FollowProtocols | 0 |  |  |  | 0.643 |
| Strongly agree |  | 11 (27%) | 7 (30%) | 4 (24%) |  |
| Agree |  | 19 (39%) | 8 (39%) | 11 (38%) |  |
| Neither agree nor disagree |  | 13 (24%) | 7 (23%) | 6 (26%) |  |
| Disagree |  | 2 (5.2%) | 1 (3.3%) | 1 (7.2%) |  |
| Strongly disagree |  | 2 (4.6%) | 1 (4.5%) | 1 (4.8%) |  |
| DistanceImportant2 | 0 |  |  |  | 0.095 |
| Agree |  | 37 (78%) | 17 (69%) | 20 (88%) |  |
| Neither agree nor disagree |  | 3 (4.9%) | 2 (7.1%) | 1 (2.4%) |  |
| Disagree |  | 7 (17%) | 5 (24%) | 2 (9.6%) |  |
| MaskImportant2 | 0 |  |  |  | 0.220 |
| Agree |  | 37 (79%) | 17 (72%) | 20 (86%) |  |
| Neither agree nor disagree |  | 5 (11%) | 3 (12%) | 2 (9.6%) |  |
| Disagree |  | 5 (11%) | 4 (16%) | 1 (4.8%) |  |
| TravelImportant2 | 1 |  |  |  | 0.986 |
| Agree |  | 29 (67%) | 15 (66%) | 14 (69%) |  |
| Neither agree nor disagree |  | 10 (19%) | 6 (23%) | 4 (14%) |  |
| Disagree |  | 7 (14%) | 3 (10%) | 4 (18%) |  |
| FollowProtocols2 | 0 |  |  |  | 0.675 |
| Agree |  | 30 (66%) | 15 (69%) | 15 (62%) |  |
| Neither agree nor disagree |  | 13 (24%) | 7 (23%) | 6 (26%) |  |
| Disagree |  | 4 (9.8%) | 2 (7.8%) | 2 (12%) |  |
|  |  |  |  |  |  |
| --- | --- | --- | --- | --- | --- |
| 1 n unweighted (% weighted) | | | | | |
| 2 Wilcoxon rank-sum test for complex survey samples | | | | | |
