# Supplementary material for: Prevalence of SARS-CoV-2 infection and immunity in a New York county in 2022 reveals frequent asymptomatic or undiagnosed infections
Source: PLoS One. 2025 May 28;20(5):e0323659. doi: 10.1371/journal.pone.0323659 (PMC12118914; doi:10.1371/journal.pone.0323659)
Supplement: S18 Table — Table of the univariate comparisons between antibody presence and behavior risk factors for infection in October 2022. (HTML) [file pone.0323659.s018.html]

| **Characteristic** | **N Missing** | **Overall** N=471 | **FALSE** N=241 | **TRUE** N=231 | **p-value**2 |
| --- | --- | --- | --- | --- | --- |
| Smoking | 0 |  |  |  | 0.339 |
| current smoker |  | 4 (11%) | 2 (7.8%) | 2 (14%) |  |
| former smoker |  | 13 (28%) | 10 (39%) | 3 (17%) |  |
| never smoked |  | 30 (61%) | 12 (53%) | 18 (69%) |  |
| Smoking2 | 0 |  |  |  | 0.367 |
|  |  | 0 (0%) | 0 (0%) | 0 (0%) |  |
| current or former smoker |  | 17 (39%) | 12 (47%) | 5 (31%) |  |
| never smoked |  | 30 (61%) | 12 (53%) | 18 (69%) |  |
| Vaping | 0 |  |  |  | 0.608 |
| current user |  | 1 (2.3%) | 0 (0%) | 1 (4.8%) |  |
| former user |  | 1 (2.3%) | 0 (0%) | 1 (4.8%) |  |
| never used a vaping device |  | 45 (95%) | 24 (100%) | 21 (90%) |  |
| Vaping2 | 0 |  |  |  | 0.326 |
|  |  | 0 (0%) | 0 (0%) | 0 (0%) |  |
| current or former vaper |  | 2 (4.6%) | 0 (0%) | 2 (9.6%) |  |
| never used a vaping device |  | 45 (95%) | 24 (100%) | 21 (90%) |  |
| Masking | 0 |  |  |  | 0.551 |
| I don't go to public places |  | 0 (0%) | 0 (0%) | 0 (0%) |  |
| Always |  | 6 (18%) | 5 (26%) | 1 (9.6%) |  |
| More than half of the time |  | 7 (13%) | 5 (17%) | 2 (9.0%) |  |
| About half of the time |  | 6 (10%) | 1 (1.7%) | 5 (20%) |  |
| Less than half of the time |  | 19 (41%) | 9 (38%) | 10 (45%) |  |
| Never |  | 9 (17%) | 4 (18%) | 5 (17%) |  |
| Masking2 | 0 |  |  |  | 0.541 |
| Less than half of the time |  | 28 (58%) | 13 (56%) | 15 (61%) |  |
| About half of the time |  | 6 (10%) | 1 (1.7%) | 5 (20%) |  |
| More than half of the time |  | 13 (31%) | 10 (43%) | 3 (19%) |  |
| MaskType | 0 |  |  |  | 0.092 |
| Cloth |  | 3 (8.1%) | 1 (2.2%) | 2 (14%) |  |
| Cloth,Other (describe in box): |  | 0 (0%) | 0 (0%) | 0 (0%) |  |
| Cloth,Respirator (such as N95, KN95, FFP2, KF94) |  | 0 (0%) | 0 (0%) | 0 (0%) |  |
| Cloth,Surgical/Medical |  | 3 (7.2%) | 2 (11%) | 1 (2.9%) |  |
| Cloth,Surgical/Medical,Other (describe in box): |  | 0 (0%) | 0 (0%) | 0 (0%) |  |
| Cloth,Surgical/Medical,Respirator (such as N95, KN95, FFP2, KF94) |  | 0 (0%) | 0 (0%) | 0 (0%) |  |
| I never wear a mask |  | 2 (4.6%) | 2 (8.9%) | 0 (0%) |  |
| Respirator (such as N95, KN95, FFP2, KF94) |  | 11 (20%) | 7 (27%) | 4 (12%) |  |
| Surgical/Medical |  | 26 (56%) | 11 (48%) | 15 (63%) |  |
| Surgical/Medical,Other (describe in box): |  | 0 (0%) | 0 (0%) | 0 (0%) |  |
| Surgical/Medical,Respirator (such as N95, KN95, FFP2, KF94) |  | 2 (4.9%) | 1 (2.7%) | 1 (7.2%) |  |
| MaskType2 | 0 |  |  |  | 0.607 |
| I never wear a mask |  | 2 (4.6%) | 2 (8.9%) | 0 (0%) |  |
| Cloth |  | 3 (8.1%) | 1 (2.2%) | 2 (14%) |  |
| Surgical/Medical |  | 29 (63%) | 13 (59%) | 16 (66%) |  |
| Respirator |  | 13 (25%) | 8 (29%) | 5 (19%) |  |
| Distancing | 0 |  |  |  | 0.108 |
| I don't go to public places |  | 0 (0%) | 0 (0%) | 0 (0%) |  |
| Always |  | 6 (13%) | 6 (25%) | 0 (0%) |  |
| More than half of the time |  | 9 (23%) | 5 (21%) | 4 (25%) |  |
| About half of the time |  | 5 (10%) | 3 (16%) | 2 (4.7%) |  |
| Less than half of the time |  | 18 (36%) | 7 (25%) | 11 (48%) |  |
| Never |  | 9 (18%) | 3 (13%) | 6 (22%) |  |
| Distancing2 | 0 |  |  |  | 0.122 |
| Less than half of the time |  | 27 (54%) | 10 (39%) | 17 (70%) |  |
| About half of the time |  | 5 (10%) | 3 (16%) | 2 (4.7%) |  |
| More than half of the time |  | 15 (36%) | 11 (46%) | 4 (25%) |  |
| Bus | 0 |  |  |  | 0.807 |
| not at all |  | 42 (92%) | 22 (92%) | 20 (91%) |  |
| once |  | 1 (1.7%) | 1 (3.3%) | 0 (0%) |  |
| twice |  | 1 (2.3%) | 1 (4.5%) | 0 (0%) |  |
| 3-5 times |  | 2 (3.5%) | 0 (0%) | 2 (7.2%) |  |
| 6-10 times |  | 1 (0.9%) | 0 (0%) | 1 (1.8%) |  |
| more than 10 times |  | 0 (0%) | 0 (0%) | 0 (0%) |  |
| Bus2 | 0 |  |  |  | 0.868 |
| not at all |  | 42 (92%) | 22 (92%) | 20 (91%) |  |
| 1-5 times |  | 4 (7.5%) | 2 (7.8%) | 2 (7.2%) |  |
| More than 5 times |  | 1 (0.9%) | 0 (0%) | 1 (1.8%) |  |
| Plane | 1 |  |  |  | 0.658 |
| not at all |  | 44 (96%) | 22 (95%) | 22 (98%) |  |
| once |  | 1 (1.2%) | 0 (0%) | 1 (2.4%) |  |
| twice |  | 0 (0%) | 0 (0%) | 0 (0%) |  |
| 3-5 times |  | 0 (0%) | 0 (0%) | 0 (0%) |  |
| 6-10 times |  | 1 (2.4%) | 1 (4.7%) | 0 (0%) |  |
| more than 10 times |  | 0 (0%) | 0 (0%) | 0 (0%) |  |
| Plane2 | 1 |  |  |  | 0.658 |
| not at all |  | 44 (96%) | 22 (95%) | 22 (98%) |  |
| 1-5 times |  | 1 (1.2%) | 0 (0%) | 1 (2.4%) |  |
| More than 5 times |  | 1 (2.4%) | 1 (4.7%) | 0 (0%) |  |
| Train | 1 |  |  |  | 0.673 |
| not at all |  | 44 (96%) | 22 (95%) | 22 (98%) |  |
| once |  | 0 (0%) | 0 (0%) | 0 (0%) |  |
| twice |  | 2 (3.6%) | 1 (4.7%) | 1 (2.4%) |  |
| 3-5 times |  | 0 (0%) | 0 (0%) | 0 (0%) |  |
| 6-10 times |  | 0 (0%) | 0 (0%) | 0 (0%) |  |
| more than 10 times |  | 0 (0%) | 0 (0%) | 0 (0%) |  |
| Train2 | 1 |  |  |  | 0.673 |
| not at all |  | 44 (96%) | 22 (95%) | 22 (98%) |  |
| 1-5 times |  | 2 (3.6%) | 1 (4.7%) | 1 (2.4%) |  |
| More than 5 times |  | 0 (0%) | 0 (0%) | 0 (0%) |  |
| Metro | 2 |  |  |  | 0.349 |
| not at all |  | 44 (99%) | 22 (100%) | 22 (98%) |  |
| once |  | 0 (0%) | 0 (0%) | 0 (0%) |  |
| twice |  | 1 (1.2%) | 0 (0%) | 1 (2.4%) |  |
| 3-5 times |  | 0 (0%) | 0 (0%) | 0 (0%) |  |
| 6-10 times |  | 0 (0%) | 0 (0%) | 0 (0%) |  |
| more than 10 times |  | 0 (0%) | 0 (0%) | 0 (0%) |  |
| Metro2 | 2 |  |  |  | 0.349 |
| not at all |  | 44 (99%) | 22 (100%) | 22 (98%) |  |
| 1-5 times |  | 1 (1.2%) | 0 (0%) | 1 (2.4%) |  |
| More than 5 times |  | 0 (0%) | 0 (0%) | 0 (0%) |  |
| Cab | 2 |  |  |  | 0.168 |
| not at all |  | 43 (95%) | 20 (90%) | 23 (100%) |  |
| once |  | 2 (4.9%) | 2 (10%) | 0 (0%) |  |
| twice |  | 0 (0%) | 0 (0%) | 0 (0%) |  |
| 3-5 times |  | 0 (0%) | 0 (0%) | 0 (0%) |  |
| 6-10 times |  | 0 (0%) | 0 (0%) | 0 (0%) |  |
| more than 10 times |  | 0 (0%) | 0 (0%) | 0 (0%) |  |
| Cab2 | 2 |  |  |  | 0.168 |
| not at all |  | 43 (95%) | 20 (90%) | 23 (100%) |  |
| 1-5 times |  | 2 (4.9%) | 2 (10%) | 0 (0%) |  |
| More than 5 times |  | 0 (0%) | 0 (0%) | 0 (0%) |  |
| Travel | 0 |  |  |  | 0.489 |
| A neighboring county in NY State |  | 15 (28%) | 8 (26%) | 7 (29%) |  |
| A neighboring county in NY State,Another State |  | 1 (1.4%) | 0 (0%) | 1 (2.9%) |  |
| A neighboring county in NY State,Somewhere else in NY State (not a neighboring county) |  | 2 (5.8%) | 2 (11%) | 0 (0%) |  |
| A neighboring county in NY State,Somewhere else in NY State (not a neighboring county),Another State,International |  | 0 (0%) | 0 (0%) | 0 (0%) |  |
| Another State |  | 7 (18%) | 3 (18%) | 4 (17%) |  |
| I have not traveled outside Tompkins County |  | 15 (34%) | 9 (38%) | 6 (30%) |  |
| International |  | 0 (0%) | 0 (0%) | 0 (0%) |  |
| Somewhere else in NY State (not a neighboring county) |  | 6 (12%) | 2 (7.0%) | 4 (17%) |  |
| Somewhere else in NY State (not a neighboring county),Another State |  | 1 (1.7%) | 0 (0%) | 1 (3.6%) |  |
| Travel2 | 0 |  |  |  | 0.885 |
|  |  | 0 (0%) | 0 (0%) | 0 (0%) |  |
| A neighboring county in NY State |  | 15 (28%) | 8 (26%) | 7 (29%) |  |
| Another State |  | 9 (21%) | 3 (18%) | 6 (24%) |  |
| I have not traveled outside Tompkins County |  | 15 (34%) | 9 (38%) | 6 (30%) |  |
| International |  | 0 (0%) | 0 (0%) | 0 (0%) |  |
| Somewhere else in NY State (not a neighboring county) |  | 8 (17%) | 4 (18%) | 4 (17%) |  |
| Gathering | 0 | 26 (51%) | 11 (45%) | 15 (58%) | 0.381 |
| N\_Gathering | 22 |  |  |  | 0.546 |
| Mean (SE) |  | 2.84 (0.43) | 2.74 (0.42) | 2.92 (0.78) |  |
| Median (IQR) |  | 2.00 (2.00, 4.00) | 2.00 (2.00, 3.96) | 2.00 (1.00, 3.43) |  |
| LargeEvent | 0 | 11 (23%) | 4 (18%) | 7 (29%) | 0.512 |
| Gym | 0 |  |  |  | 0.914 |
| 0 |  | 39 (81%) | 20 (82%) | 19 (80%) |  |
| 1 - 3 times |  | 6 (13%) | 3 (12%) | 3 (15%) |  |
| 10 - 12 times |  | 0 (0%) | 0 (0%) | 0 (0%) |  |
| 4 - 6 times |  | 2 (5.8%) | 1 (6.7%) | 1 (4.8%) |  |
| 7 - 9 times |  | 0 (0%) | 0 (0%) | 0 (0%) |  |
| more than 12 times |  | 0 (0%) | 0 (0%) | 0 (0%) |  |
| Gym2 | 0 |  |  |  | 0.870 |
|  |  | 0 (0%) | 0 (0%) | 0 (0%) |  |
| 0 |  | 39 (81%) | 20 (82%) | 19 (80%) |  |
| 1 - 6 times |  | 8 (19%) | 4 (18%) | 4 (20%) |  |
| More than 6 times |  | 0 (0%) | 0 (0%) | 0 (0%) |  |
| IndoorDining | 0 |  |  |  | 0.412 |
| 0 |  | 19 (39%) | 11 (45%) | 8 (32%) |  |
| 1 - 3 times |  | 23 (54%) | 11 (48%) | 12 (61%) |  |
| 4 - 6 times |  | 3 (3.9%) | 1 (2.7%) | 2 (5.3%) |  |
| 7 - 9 times |  | 1 (0.9%) | 0 (0%) | 1 (1.8%) |  |
| More than 9 times |  | 1 (2.3%) | 1 (4.5%) | 0 (0%) |  |
| IndoorDining2 | 0 |  |  |  | 0.399 |
|  |  | 0 (0%) | 0 (0%) | 0 (0%) |  |
| 0 |  | 19 (39%) | 11 (45%) | 8 (32%) |  |
| 1 - 3 times |  | 23 (54%) | 11 (48%) | 12 (61%) |  |
| More than 3 times |  | 5 (7.1%) | 2 (7.1%) | 3 (7.1%) |  |
| HandWashing | 0 |  |  |  | 0.830 |
| Decreased this behavior |  | 0 (0%) | 0 (0%) | 0 (0%) |  |
| Haven't changed |  | 8 (11%) | 4 (10%) | 4 (12%) |  |
| Increased this behavior |  | 39 (89%) | 20 (90%) | 19 (88%) |  |
| HandSanitizer | 0 |  |  |  | 0.583 |
| Decreased this behavior |  | 1 (1.2%) | 1 (2.2%) | 0 (0%) |  |
| Haven't changed |  | 13 (22%) | 7 (24%) | 6 (19%) |  |
| Increased this behavior |  | 33 (77%) | 16 (73%) | 17 (81%) |  |
| TouchingFace | 0 |  |  |  | 0.658 |
| Decreased this behavior |  | 15 (36%) | 8 (38%) | 7 (33%) |  |
| Haven't changed |  | 31 (63%) | 16 (62%) | 15 (65%) |  |
| Increased this behavior |  | 1 (0.9%) | 0 (0%) | 1 (1.8%) |  |
| Cleaning | 0 |  |  |  | 0.273 |
| Decreased this behavior |  | 0 (0%) | 0 (0%) | 0 (0%) |  |
| Haven't changed |  | 21 (38%) | 13 (47%) | 8 (29%) |  |
| Increased this behavior |  | 26 (62%) | 11 (53%) | 15 (71%) |  |
| StayHomeSick | 0 |  |  |  | 0.064 |
| Decreased this behavior |  | 1 (1.4%) | 1 (2.7%) | 0 (0%) |  |
| Haven't changed |  | 21 (43%) | 8 (29%) | 13 (59%) |  |
| Increased this behavior |  | 25 (56%) | 15 (69%) | 10 (41%) |  |
| Doctors | 1 |  |  |  | 0.373 |
| Decreased this behavior |  | 2 (3.8%) | 2 (7.3%) | 0 (0%) |  |
| Haven't changed |  | 39 (85%) | 17 (72%) | 22 (98%) |  |
| Increased this behavior |  | 5 (11%) | 4 (21%) | 1 (1.8%) |  |
| NursingHome | 0 |  |  |  | 0.740 |
| Decreased this behavior |  | 2 (5.8%) | 1 (4.5%) | 1 (7.2%) |  |
| Haven't changed |  | 43 (90%) | 22 (91%) | 21 (89%) |  |
| Increased this behavior |  | 2 (4.0%) | 1 (4.5%) | 1 (3.6%) |  |
| Telehealth | 1 |  |  |  | 0.388 |
| Decreased this behavior |  | 0 (0%) | 0 (0%) | 0 (0%) |  |
| Haven't changed |  | 19 (43%) | 11 (51%) | 8 (35%) |  |
| Increased this behavior |  | 27 (57%) | 12 (49%) | 15 (65%) |  |
| Curbside | 0 |  |  |  | 0.685 |
| Decreased this behavior |  | 0 (0%) | 0 (0%) | 0 (0%) |  |
| Haven't changed |  | 27 (56%) | 14 (59%) | 13 (52%) |  |
| Increased this behavior |  | 20 (44%) | 10 (41%) | 10 (48%) |  |
|  |  |  |  |  |  |
| --- | --- | --- | --- | --- | --- |
| 1 n unweighted (% weighted) | | | | | |
| 2 Wald test of independence for complex survey samples; Wilcoxon rank-sum test for complex survey samples; Kruskal-Wallis rank-sum test for complex survey samples | | | | | |
