# Supplementary material for: Prevalence of SARS-CoV-2 infection and immunity in a New York county in 2022 reveals frequent asymptomatic or undiagnosed infections
Source: PLoS One. 2025 May 28;20(5):e0323659. doi: 10.1371/journal.pone.0323659 (PMC12118914; doi:10.1371/journal.pone.0323659)
Supplement: S19 Table — Table of the univariate comparisons between antibody presence and demographic risk factors for infection in October 2022. (HTML) [file pone.0323659.s019.html]

| **Characteristic** | **N Missing** | **Overall** N=471 | **FALSE** N=241 | **TRUE** N=231 | **p-value**2 |
| --- | --- | --- | --- | --- | --- |
| Age | 0 |  |  |  | 0.625 |
| Mean (SE) |  | 52 (4) | 53 (5) | 51 (4) |  |
| Median (IQR) |  | 55 (34, 66) | 57 (34, 68) | 53 (34, 64) |  |
| Age2 | 0 |  |  |  | 0.418 |
| 18 to 64 |  | 30 (67%) | 14 (63%) | 16 (71%) |  |
| 65 and over |  | 17 (33%) | 10 (37%) | 7 (29%) |  |
| Under 18 |  | 0 (0%) | 0 (0%) | 0 (0%) |  |
| Gender | 0 |  |  |  | 0.589 |
| Female |  | 27 (56%) | 13 (52%) | 14 (59%) |  |
| Gender non-binary |  | 1 (2.3%) | 0 (NA%) | 1 (NA%) |  |
| Male |  | 19 (42%) | 11 (48%) | 8 (36%) |  |
| Other |  | 0 (0%) | 0 (0%) | 0 (0%) |  |
| Gender2 | 0 |  |  |  | 0.589 |
|  |  | 0 (0%) | 0 (0%) | 0 (0%) |  |
| Female |  | 27 (56%) | 13 (52%) | 14 (59%) |  |
| Male |  | 19 (42%) | 11 (48%) | 8 (36%) |  |
| Other |  | 1 (2.3%) | 0 (0%) | 1 (4.8%) |  |
| Race | 2 |  |  |  | 0.239 |
| American Indian or Alaskan Native |  | 1 (2.4%) | 0 (0%) | 1 (4.9%) |  |
| American Indian or Alaskan Native,Native Hawaiian or Pacific Islander |  | 0 (0%) | 0 (0%) | 0 (0%) |  |
| American Indian or Alaskan Native,White |  | 0 (0%) | 0 (0%) | 0 (0%) |  |
| Asian |  | 2 (3.3%) | 0 (0%) | 2 (6.7%) |  |
| Asian,White |  | 0 (0%) | 0 (0%) | 0 (0%) |  |
| Black or African American |  | 0 (0%) | 0 (0%) | 0 (0%) |  |
| White |  | 42 (94%) | 23 (100%) | 19 (88%) |  |
| Race2 | 0 |  |  |  | 0.305 |
|  |  | 2 (3.2%) | 1 (4.5%) | 1 (1.8%) |  |
| American Indian or Alaskan Native |  | 1 (2.3%) | 0 (0%) | 1 (4.8%) |  |
| Asian |  | 2 (3.2%) | 0 (0%) | 2 (6.6%) |  |
| Black or African American |  | 0 (0%) | 0 (0%) | 0 (0%) |  |
| Two or more races |  | 0 (0%) | 0 (0%) | 0 (0%) |  |
| White |  | 42 (91%) | 23 (96%) | 19 (87%) |  |
| Ethnicity | 2 |  |  |  | 0.860 |
| Hispanic or Latino |  | 2 (4.8%) | 1 (4.6%) | 1 (4.9%) |  |
| Not Hispanic or Latino |  | 43 (95%) | 22 (95%) | 21 (95%) |  |
| Education | 0 |  |  |  | 0.853 |
| Associate's Degree |  | 7 (16%) | 4 (20%) | 3 (11%) |  |
| Bachelor's Degree |  | 9 (17%) | 5 (19%) | 4 (14%) |  |
| Between grades 9 - 11 |  | 0 (0%) | 0 (0%) | 0 (0%) |  |
| Graduate/ Professional School Degree |  | 24 (52%) | 11 (47%) | 13 (57%) |  |
| High School Diploma / GED certificate |  | 5 (11%) | 3 (11%) | 2 (11%) |  |
| none or between grades 1 - 8 |  | 0 (0%) | 0 (0%) | 0 (0%) |  |
| Technical, trade or vocational school |  | 2 (4.6%) | 1 (2.2%) | 1 (7.2%) |  |
| Education2 | 14 |  |  |  | 0.541 |
| No Bachelor's Degree |  | 0 (0%) | 0 (0%) | 0 (0%) |  |
| Bachelor's Degree |  | 9 (24%) | 5 (29%) | 4 (19%) |  |
| Graduate/ Professional School Degree |  | 24 (76%) | 11 (71%) | 13 (81%) |  |
| HH Size | 1 |  |  |  | 0.343 |
| Mean (SE) |  | 2.11 (0.17) | 2.03 (0.30) | 2.19 (0.14) |  |
| Median (IQR) |  | 2.00 (2.00, 2.00) | 2.00 (1.00, 2.00) | 2.00 (2.00, 2.00) |  |
| Number of Generations in HH | 14 |  |  |  | 0.165 |
| 1 |  | 23 (72%) | 9 (59%) | 14 (85%) |  |
| 2 |  | 9 (25%) | 6 (41%) | 3 (11%) |  |
| 3 |  | 1 (2.2%) | 0 (0%) | 1 (4.2%) |  |
|  |  |  |  |  |  |
| --- | --- | --- | --- | --- | --- |
| 1 n unweighted (% weighted) | | | | | |
| 2 Kruskal-Wallis rank-sum test for complex survey samples; Wald test of independence for complex survey samples | | | | | |
