# Supplementary material for: Prevalence of SARS-CoV-2 infection and immunity in a New York county in 2022 reveals frequent asymptomatic or undiagnosed infections
Source: PLoS One. 2025 May 28;20(5):e0323659. doi: 10.1371/journal.pone.0323659 (PMC12118914; doi:10.1371/journal.pone.0323659)
Supplement: S20 Table — Table of the univariate comparisons between antibody presence and work risk factors for infection in October 2022. (HTML) [file pone.0323659.s020.html]

| **Characteristic** | **N Missing** | **Overall** N=471 | **FALSE** N=241 | **TRUE** N=231 | **p-value**2 |
| --- | --- | --- | --- | --- | --- |
| Employment | 0 |  |  |  |  |
| Disabled |  | 2 (5.2%) | 2 (10%) | 0 (0%) |  |
| Full time employment |  | 24 (49%) | 12 (46%) | 12 (52%) |  |
| Full time employment,Self-employed |  | 1 (0.9%) | 0 (0%) | 1 (1.8%) |  |
| Full time employment,Student |  | 0 (0%) | 0 (0%) | 0 (0%) |  |
| Part time employment |  | 1 (1.7%) | 0 (0%) | 1 (3.6%) |  |
| Part time employment,Self-employed |  | 1 (0.9%) | 0 (0%) | 1 (1.8%) |  |
| Part time employment,Student |  | 0 (0%) | 0 (0%) | 0 (0%) |  |
| Retired |  | 12 (26%) | 8 (33%) | 4 (19%) |  |
| Self-employed |  | 3 (6.9%) | 1 (6.7%) | 2 (7.2%) |  |
| Student |  | 2 (4.6%) | 1 (4.5%) | 1 (4.8%) |  |
| Unemployed |  | 1 (4.6%) | 0 (0%) | 1 (9.6%) |  |
| Employment2 | 0 |  |  |  | 0.637 |
|  |  | 0 (0%) | 0 (0%) | 0 (0%) |  |
| Employed |  | 30 (59%) | 13 (53%) | 17 (66%) |  |
| Retired, Unemployed, or Disabled |  | 15 (36%) | 10 (43%) | 5 (29%) |  |
| Student |  | 2 (4.6%) | 1 (4.5%) | 1 (4.8%) |  |
| WorkLocation | 17 |  |  |  | 0.086 |
| Other (describe): |  | 0 (0%) | 0 (0%) | 0 (0%) |  |
| All remote work |  | 3 (8.5%) | 1 (10%) | 2 (7.1%) |  |
| Mostly remote (more than half) with some in-person work |  | 4 (16%) | 4 (34%) | 0 (0%) |  |
| About half in-person and half remote |  | 4 (14%) | 2 (12%) | 2 (16%) |  |
| Mostly in-person (more than half) with some remote work |  | 2 (4.4%) | 0 (0%) | 2 (8.2%) |  |
| All in-person (on-site) work |  | 17 (57%) | 6 (45%) | 11 (68%) |  |
| WorkLocation2 | 36 |  |  |  | 0.129 |
| More than half remote |  | 7 (63%) | 5 (79%) | 2 (30%) |  |
| About half in-person and half remote |  | 4 (37%) | 2 (21%) | 2 (70%) |  |
| More than half in person |  | 0 (0%) | 0 (0%) | 0 (0%) |  |
| WorkNumContact | 20 |  |  |  | 0.491 |
| 0 to 5 |  | 13 (48%) | 7 (56%) | 6 (42%) |  |
| 6 to 10 |  | 6 (19%) | 1 (9.4%) | 5 (27%) |  |
| 11 to 20 |  | 5 (20%) | 3 (29%) | 2 (12%) |  |
| More than 20 |  | 3 (13%) | 1 (5.6%) | 2 (19%) |  |
| WorkNumContact2 | 20 |  |  |  | 0.833 |
| 0 to 10 |  | 19 (67%) | 8 (65%) | 11 (69%) |  |
| More than 10 |  | 8 (33%) | 4 (35%) | 4 (31%) |  |
| WorkPublic | 20 | 14 (52%) | 5 (44%) | 9 (58%) | 0.438 |
|  |  |  |  |  |  |
| --- | --- | --- | --- | --- | --- |
| 1 n unweighted (% weighted) | | | | | |
| 2 Wald test of independence for complex survey samples; Wilcoxon rank-sum test for complex survey samples | | | | | |
