# Supplementary material for: Prevalence of SARS-CoV-2 infection and immunity in a New York county in 2022 reveals frequent asymptomatic or undiagnosed infections
Source: PLoS One. 2025 May 28;20(5):e0323659. doi: 10.1371/journal.pone.0323659 (PMC12118914; doi:10.1371/journal.pone.0323659)
Supplement: Attachment — Submitted filename: Response to reviewers.docx [file pone.0323659.s022.docx]

Reviewer #1: A review of the manuscript entitled “Prevalence of SARS-CoV-2 infection and immunity in a New York county in 2022 reveals frequent asymptomatic or undiagnosed infections”

1. (page 5, lines 48-49): This sentence “Disease surveillance is a cornerstone of public health practice, particularly to understand and control the spread of infectious pathogens” will be more significant if it’s also supported by another relevant study. Please check “Global prevalence of persistent neuromuscular symptoms and the possible pathomechanisms in COVID-19 recovered individuals: A systematic review and meta-analysis”

***Authors****: The sentence is intentionally broad and not specific to COVID-19, hence we did not cite a COVID-19 paper but rather an overview of public health surveillance. We decline to include this reference in this section.*

2. (page 11, lines 147-150): The sentence also shares the same idea with a study entitled “Comparison of RT-PCR cycle threshold values between individual and pooled SARS-CoV-2 infected nasopharyngeal swab specimens” Kindly include this reference to make the sentence stronger.

***Authors:*** *The reference we included (Laverack M, Tallmadge RL, Venugopalan R, Cronk B, Zhang X, Rauh R, et al. Clinical evaluation of a multiplex real-time RT-PCR assay for detection of SARS-CoV-2 in individual and pooled upper respiratory tract samples. Arch Virol. 2021;166(9):2551-61) was a description of the actual test used in our study. We do not want to confuse readers and cite an assay that we did not use. Therefore, we decline to include this reference here.*

3. (page 14, lines 216-218): The authors stated that “Our sample population included slightly more females, older individuals, and white individuals than all three communities.” My question is what are those three communities? Please briefly mention them in this sentence.

***Authors:*** *The specific communities were added (line 223).*

4. (page 16, lines 223-224): The sentence has a few grammatical issues that need correction. Please consider omitting the “were positive” part to make the sentence clear. Thus, the final sentence looks like this “In the City of Ithaca in February 2022, only two out of 105 samples on SARS-CoV-2 PCR testing was positive (test positivity of 1.9%).”

***Authors:*** *This sentence was corrected (line 229).*

5. Overall, this manuscript has shown a sophisticated review of the estimation of SARS-CoV-2 prevalence in Tompkins County, using lots of relevant references in related areas. The use of good English has also been applied throughout the manuscript. However, I have some minor comments, as already mentioned above, to increase the clarity of this review manuscript. Thank you.

Reviewer #2: Thank you for the opportunity to review this well-written manuscript. The study by Cazer et al. estimated prevalence of COVID-19 infection from population-representative household surveys in Tompkins County. The authors note that the use of indicators such as percent positivity from case based surveillance are biased and are likely underestimating the true prevalence of infection in the community. The study sampled households in three municipalities at one time point for each.

In summary, the study found that the prevalence of active infection was less than 5%, about half of the population had a prior infection, and a large proportion of survey participants had hybrid immunity due to either prior infection of vaccination. Authors note substantial proportion of likely asymptomatic infections and waning immunity post-vaccination and post-infection over time especially in the survey administered in Oct 2022. Stated strengths include probability-based sampling, and weaknesses include small sample size that precluded analyses looking at behaviors and previous and current infections.

Main comments:
1. While probability-based sampling in population representative surveys can reduce bias, population representative surveys are not completely impervious to selection bias which can occur at different stages of sampling and inclusion (i.e., who is invited and who responds/participates in survey). Limitations on the possible sources of bias should be transparent and included in the discussion. For example, it is important to note that the HU sampling scheme excludes group homes, dormitories and assisted living facilities, which limits the external validity of the findings especially when compared to cases reported through surveillance (which presumably captures cases from those places). Further, non-response bias may have occurred among those who agreed to consent to the survey. If those who responded are more or less likely to have been previously infected or previously vaccinated, then it could affect your prevalence estimates. The directionality of the bias could either over- or under-estimate the prevalence but noting this potential source of bias is important in the discussion.

***Authors:*** *A discussion of selection bias and non-response bias has been added (lines 346-359).*

2.Was there information available on those who declined participation? If so, were they compared with those who chose to participate? How were they different?

***Authors:*** *There is no information available on individuals who declined to participate, had “no solicitation signs”, or were not home at the time of the survey. A discussion of selection bias and non-response bias has been added (lines 346-359).*

3.Did participants get access to their test results? If so, motivations to get tested in order to obtain their test results may bias the estimates (e.g., those who were symptomatic are more likely to get tested to confirm test result, and therefore may be over-representative). Would suggest incorporating additional assessment of potential sources of selection bias in the discussion to address the points above.
***Authors:*** *A discussion of selection bias and non-response bias has been added (lines 346-359). Participants did receive their test results; this was added to line 155.*

Other:

4.Were sample size calculations done for this study? Would suggest including the decision around the number of HU per cluster sampled.

***Authors:*** *Details on the power of the study design were added to lines 109-111.*

5.The paper would benefit from a brief situation analysis of circulating variants during the survey time periods.

***Authors:*** *This information was added (lines 330-332).*

6.Introduction, line 53; would include the use of rapid at-home diagnostic testing as it would have contributed to individuals who tested at home and did not seek confirmatory testing with a provider and therefore led to an under count of cases.

***Authors:*** *This was added to line 54.*

7.Did county-level metrics only include PCR tests and test results? Some jurisdictions also include probable cases based on rapid antigen tests. If those were available, would consider presenting those numbers as well by comparing the survey-based estimates with PCR (confirmed) and rapid antigen test (probable).

***Authors:*** *The reported county-level metrics only included PCR tests. We added the PCR and rapid antigen tests to lines 254-259. We cannot use the rapid antigen tests in the positive test prevalence since the denominator of rapid tests performed is unknown.*
