# Supplementary material for: Prevalence of SARS-CoV-2 infection and immunity in a New York county in 2022 reveals frequent asymptomatic or undiagnosed infections
Source: PLoS One. 2025 May 28;20(5):e0323659. doi: 10.1371/journal.pone.0323659 (PMC12118914; doi:10.1371/journal.pone.0323659)
Supplement: Attachment — Submitted filename: Response_to_reviewers_auresp_2.docx [file pone.0323659.s023.docx]

The revised version of this manuscript should prioritize the following points:

1. Please include the limitations on the sources' bias in the discussion.

**Authors:** *A discussion of selection bias and non-response bias is in the discussion (lines 346-359). We have included the directionality of the bias (potential under-estimation of prevalence, lines 351-352 and lines 358-359). If there are additional biases that you would like to see discussed, please provide specifics.*

2. Provide information on the sample size calculation.

**Authors:** *Details on the sample size calculation and power of the study design are in lines 109-113. The sample size was calculated based on prevalence precision. The equation was added (lines 113-116).*

3. The manuscript should have the writing errors corrected.

**Authors:** The manuscript was thoroughly reviewed for typos and grammatical errors. Please advise if specific errors are identified.
