# Supplementary material for: Prevalence of SARS-CoV-2 infection and immunity in a New York county in 2022 reveals frequent asymptomatic or undiagnosed infections
Source: PLoS One. 2025 May 28;20(5):e0323659. doi: 10.1371/journal.pone.0323659 (PMC12118914; doi:10.1371/journal.pone.0323659)
Supplement: Attachment — Submitted filename: Response to Reviewers R3.docx [file pone.0323659.s024.docx]

Journal Requirements:

Please review your reference list to ensure that it is complete and correct. If you have cited papers that have been retracted, please include the rationale for doing so in the manuscript text, or remove these references and replace them with relevant current references. Any changes to the reference list should be mentioned in the rebuttal letter that accompanies your revised manuscript. If you need to cite a retracted article, indicate the article’s retracted status in the References list and also include a citation and full reference for the retraction notice.
**Authors: We confirmed that no references have been retracted and that the reference list is complete and correct.**

Reviewers' comments:

Reviewer's Responses to Questions

**Comments to the Author**

Review Comments to the Author

Please use the space provided to explain your answers to the questions above. You may also include additional comments for the author, including concerns about dual publication, research ethics, or publication ethics. (Please upload your review as an attachment if it exceeds 20,000 characters)

Reviewer #1: (No Response)

Reviewer #3: 1. On page 6 lines 72-74, I suggest that the authors add an additional group of people other than those in university settings, for example, the global survivors of COVID-19. Thus, the sentence becomes like this: “Finally, surveillance for behavioral factors that mitigate or promote COVID-19 infection risk is important in developing public health recommendations targeting groups at high risk (17-20), including in university settings (21) and the global survivors of COVID-19 (36).” The 36th reference can be cited from a study by Fahriani et al. entitled “Persistence of long COVID symptoms in COVID-19 survivors worldwide and its potential pathogenesis -A systematic review and meta-analysis”.

**Authors: Our references already include individuals in non-university settings across the globe. See:**

**18. Benham JL, Lang R, Kovacs Burns K, MacKean G, Leveille T, McCormack B, et al. Attitudes, current behaviours and barriers to public health measures that reduce COVID-19 transmission: A qualitative study to inform public health messaging. PLoS One. 2021;16(2):e0246941.**

**19. MacIntyre CR, Nguyen PY, Chughtai AA, Trent M, Gerber B, Steinhofel K, et al. Mask use, risk-mitigation behaviours and pandemic fatigue during the COVID-19 pandemic in five cities in Australia, the UK and USA: A cross-sectional survey. Int J Infect Dis. 2021;106:199-207.**

**In addition, the suggested reference does not seem relevant to the sentence as it does not address behavioral risk factors and public health recommendations. It is about long COVID symptoms and pathogenesis. Therefore, we have declined to include the reference.**

2. This sentence on page 6 lines 74-76 will be deeper if authors could provide an example of the settings they mention before, such as healthcare professional settings. Thus the sentence will look like this “Integrating surveillance for active infection with surveillance for preventive behaviors can be successful in managing COVID-19 infection in some settings (22), such as the healthcare professional settings (37).” This 37th reference can be found in a study by Hamdan et al. entitled “Coping strategies used by healthcare professionals during COVID-19 pandemic in Dubai: A descriptive cross-sectional study”.

**Authors: The suggested reference is not relevant for this sentence, as it does not discuss the integration of infection surveillance and preventive behavior surveillance. Therefore, we have declined to include the reference.**

3. On page 9 table 1, the authors stated that one of the conditions that a participant was excluded from this study was being less than 2 years old. Does it mean that authors include minors starting from 3 years old and above? Aren’t they too young to deal with a complex survey like this?

**Authors: We have clarified in sentence 122-123 that parents or guardians completed the survey for minors who could not complete it themselves, such as 2 year olds and 3 year olds.**

4. Please revise the phrase on line 156 into “…anterior nares swab samples.”

**Authors: This has been corrected.**
